# Global reform population health management as stewarded by Higher Expert Medical Science Safety (HEMSS)

**DOI:** 10.3389/frai.2025.1496948

**Published:** 2025-09-04

**Authors:** James Andrew Henry

**Affiliations:** Institute of Biomedical Sciences, London, United Kingdom

**Keywords:** memorandum of understanding on AI, population health management, predictive health, precision care, higher expert medical science safety

## Abstract

As described in a Memorandum of Understanding (MoU) on AI infrastructure, global human phenotype ontology (HPO) is a priority for the US and the UK. The UK NHS Act of 1946 and the Medicare and Medicaid Act of 1965 classify using genomics as primary care, supporting international HPO aims for Population Health Management (PHM). The Higher Expert Medical Science Safety (HEMSS) proposes the NHS England, Genomics, and Biobank agile group developers. The HEMSS strategy executes the PHM of the HPO through digital records, pilot citizen predictor pre-eXams, and precise eXam intercept classifications, continuously improving public safety. PHM reform includes biobank opportunities for Value-Based Care (VBC) stratifying genomic and socio-environmental factors that risk HPO in disease segmentation. The author evaluated a standard approach to PHM for HPO with mature and advanced interoperable standards. A reform toolkit aligns adversarial, neural, and transformer models for Generative AI by utilizing multimodal data nuanced for fairness in Quantum Intelligence. The recommendations include HEMSS steps from well-being evaluations to the PHM strategy for HPO in the UK-US. Concepts involve piloting the scaling up of neighborhood clinics and federal centers through reform classification. Plans for citizen privacy facilitate data use with access to reference biobanks, ensuring DNA democratization and national cybersecurity. The UK NHSE corporate governance and US federal authorities monitor and reform the Integrated Care Board assessments and the Centers for Medicare and Medicaid Services surveys using agile methods. The UK-US MoU for AI safety is an international ideal for PHM, creating a safe space for HPO adherence to predictive and interceptive adoption for health and socioeconomic growth. HEMSS Agile Group Development impacts ethical and societal primary care debates. HEMSS discussions on global public health inclusiveness and national engagement aim to govern the classification phases for adherence. Therefore, debates on UK-US accreditation or regulation on the future of Artificial General Intelligence follow. The author concludes in support of the Population Health Management Expert Medical Science Safety Agile Group Development Program. The UK and US governments would benefit from this proposition, and international goals for well-being and socioeconomic growth would also be supported.

## 1 Introduction

The UK’s NHS Act of 1946 and the US Medicare and Medicaid Act of 1965 laid the foundations for Population Health Management (PHM), shaping reforms for Human Phenotype Ontology (Legisltaion.Gov.UK, 1946; National Archives, 2022). The Department for Science, Innovation, and Technology (DSIT) and the National Science and Technology Council (NSCT) aim to structure knowledge in a Memorandum of Understanding (MoU) on AI for a PHM ecosystem by 2030 (UK.GOV, 2024; Department of Science, Innovation and Technology, 2023; National Science and Technology Council, 2022). Achieving the HPO ecosystem based on genomic and social factors would realize the UN’s Sustainable Development Goal 3 for Good Health and Well-being and Goal 8 for Economic Growth (United Nations, 2023; U. Environment, 2021).

**Figure 1:**
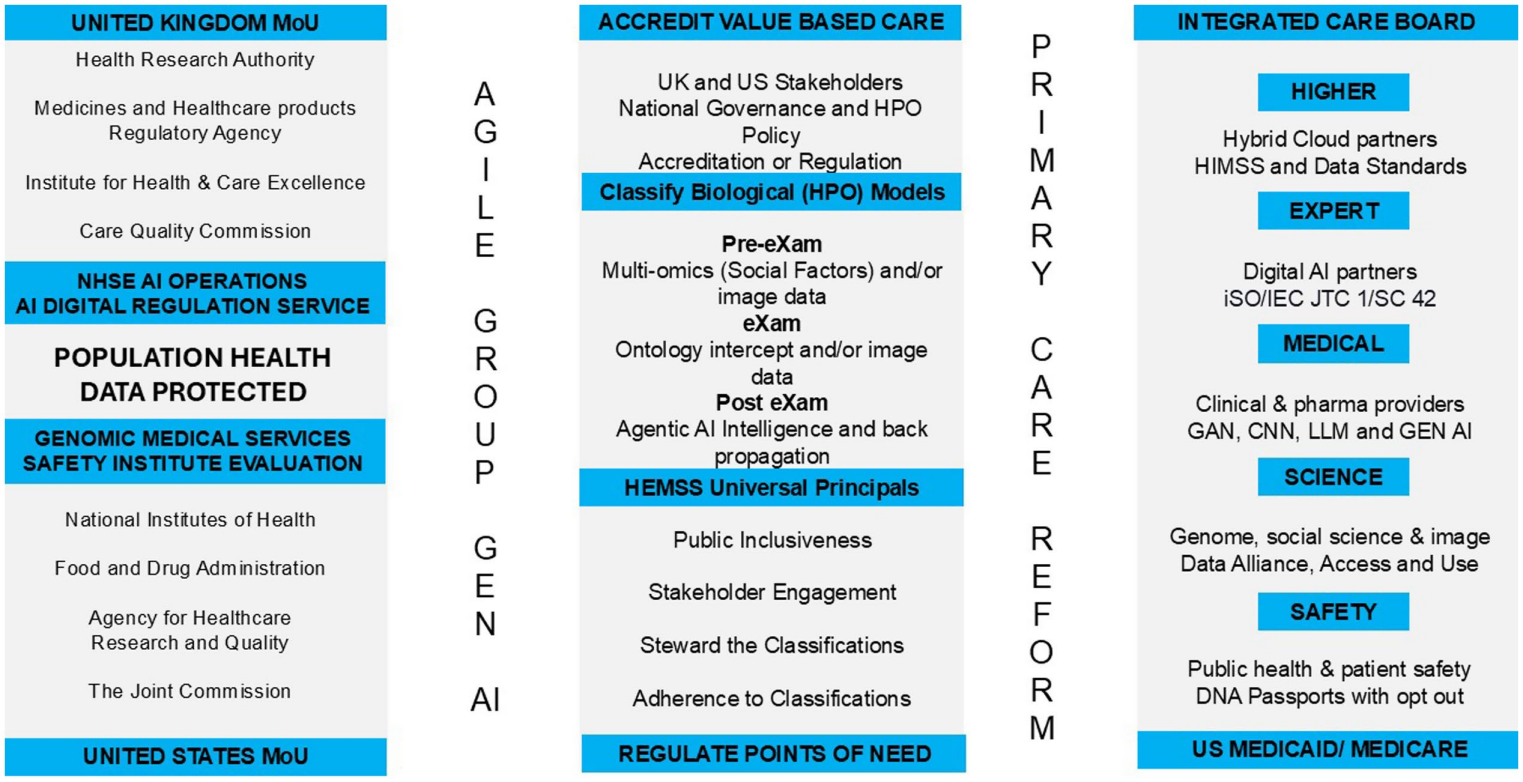
Population health management–structure higher expert medical science safety with agile group development.

Figure 1 depicts the Genomic Medical Service (GMS) overview of the HPO reform with the UK-US AI Security/Safety Institute pre-deployment tests for biological modelling (NIST, 2024). The figure illustrates that adoption would be accelerated by a Higher Expert Medical Science Safety (HEMSS) task force. NHS England (NHSE) PHM aims to integrate advanced AI with data alliances for comprehensive, equitable, and safe HPO points of need (NHS England, 2021b; UK Health Data Research Alliance, 2024; Institute of Biomedical Sciences, 2023). US Federal Care would sustain a PHM ecosystem powered by HPO, under HEMSS oversight that stratifies risk and segments disease for Agile Group Developers to classify value-based predictors and intercepts as fit-for-purpose (Crane et al., 2022; National Committee for Quality Assurance in Collaboration with Health Management Associates, 2024).

### 1.1 Background to UK and US human phenotype ontology reform

In the UK, reform has been a focus because of the increased pressure faced by the NHS, with issues such as an aging population with chronic care needs, longer waiting times, and underfunding (Alderwick et al., 2024). Simultaneously, the HEMSS offers help in moving the NHSE and GMS forward; it engages and governs valid classifications for PHM adherence. Reform would require a shift from a hospital-centric model to a patient-centric knowledge graph that verifies early prediction and precise intervention (NIST, 2024; Khatib et al., 2024). This is possible because Integrated Care Boards could replace Clinical Commissioning Groups in the UK with citizens’ digital identities (NHS England, 2021a). The infrastructure for PHM would improve public health, patient safety, and parity as a broader risk strategy with the HEMSS Agile Group Developments for HPO reform (NHSE, 2024; NHSE, 2023).

In the US, HPO reform has been less driven over the past decade, as there are significant disparities between services characterized by public and private resources with variations in access, quality, and cost of care (Sandhu et al., 2021). Nevertheless, the US has expanded Medicaid under the Affordable Care Act and has seen improvements in coverage and health outcomes in contrast with poorer health indicators in states that have not expanded Medicaid (Brown et al., 2021). The Centers for Medicare and Medicaid Services (CMS) oversee programs and implement reforms to improve Value-Based Care (VBC) access and equity in wellbeing (Donohue et al., 2022). Despite efforts to integrate ecosystem VBC, PHM-centeredness challenges authorities and commissioners (Wang et al., 2023), and the author proposes a task force on HEMSS principles, standards, and tools, as illustrated in Figure 1.

### 1.2 Foresight for a memorandum of understanding on AI

As Figure 1 shows, the UK-US MoU with AISI aims to support AGI in PHM and to develop interoperable and algorithm-driven HPO solutions for global adoption. The US Secretary of Commerce states that the two countries are laying the groundwork to ensure that they are keeping AI safe both now and in the future, while the UK Secretary of State for the DSIT aims to “deepen our enduring special relationship to address the defining technology challenge of our generation” (US Department of Commerce, 2024). A memorandum of understanding on PHM is more relevant than ever since the introduction of the UK Generation Study, which provides insight and evidence of biological models from pangenome research for integrating HPO primary care (NHS England, 2024; Liao et al., 2023), whilst HEMSS provides a “classical” approach on agile method integrations.

Figure 1 depicts corporate agile group Generative AI for primary HPO care reform: UK pilots for US ecosystems exchange PHM as predictive health pre-eXams and precise eXam intercepts which are “classically” evidence-based. Standard UK-US AI Safety Institute tools evaluate PHM practices to scale up GPU capacity and distribute them on servers throughout the US CMS and UK regions to facilitate primary care reform (Stackzone, 2024). The HEMSS public inclusivity and stakeholder engagement govern the classification of predictors and intercepts developed by the AIDRS for adoptive adherence (NHS Beta, n.d.). This manuscript program pilots “PHM HEMSS Agile Group Development” for UK Integrated Care Board (ICB) clinics and US Centers for Medicare and Medicaid Services (CMS) as a strategy for VBC (Figure 1).

### 1.3 Global population health

Global population health management aims requires a lifelong assessment of human phenotype ontology that action biological modelling from multiple perspectives to a unified ecosystem through sections 1.3.1 to 1.3.22.

#### 1.3.1 International aims for population health management

The international PHM aims to achieve universal health coverage and access to quality healthcare by 2030, as the WHO and UN Sustainable Development Goals execute an agenda that emphasizes the importance of ecosystem strengthening to achieve targets (World Health Organization, 2023). The US Healthy People 2030 and UK People Plan set national aims to improve health and well-being (Office of Disease Prevention and Health Promotion, 2024; NHS England, 2020). However, reforming the PHM transformation infrastructure performance program by 2030 using AI policy for UK-US healthcare reform presents challenges, which HEMSS principles address to accelerate “classical” HPO in both countries (Infrastructure and Projects Authority, 2021; Center for AI and Digital Policy, n.d.).

**Figure 2:**
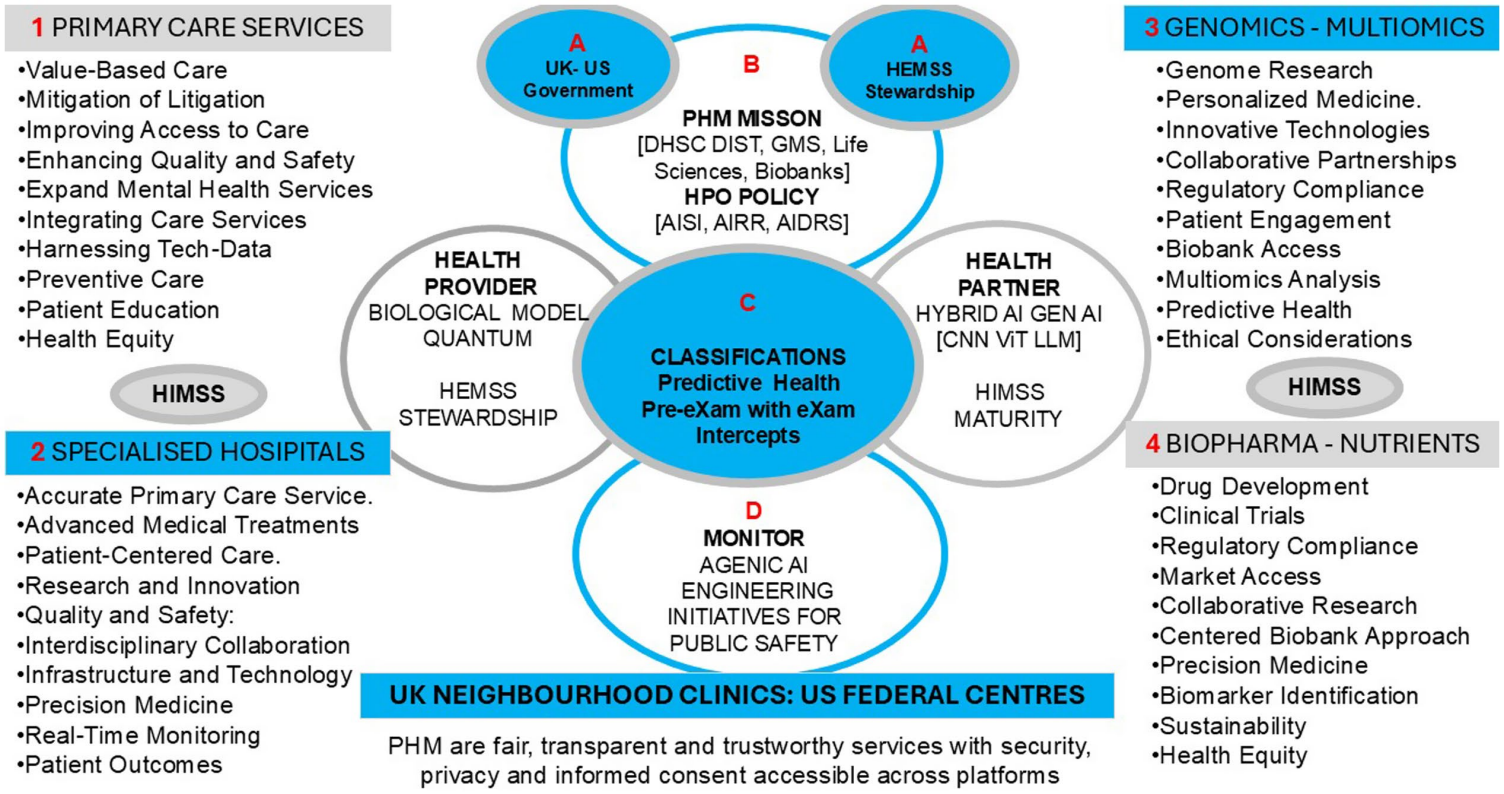
Population health management policy for UK clinics and US centers for value base are care at the point of need.

Figure 2 provides an overview of the strategic proposal with the strategy for “Population Health Management Higher Expert Medical Science Safety Agile Group Development” as the perspective for HPO risk stratification to predict wellbeing and action disease segmentation for target intercepts by engineering public safety. These are presented in Section 1.3.2 of the US and UK AI strategy for Population Health Management and Section 1.3.3 of the Higher Expert Medical Science Safety Agile Group Development. In Section 1.3.4, HPO outlines predictor pre-eXams and precise care eXams classification, and Section 1.3.5 contains Ontology System Engineering Initiatives for Public Safety.

#### 1.3.2 UK-US AI policy for population health management

The US Center for AI and Digital Policy (CAIDP) may achieve a better society by ensuring that technology promotes broad social inclusion through fundamental rights, democratic institutions, and the rule of law (Center for AI and Digital Policy, n.d.). Executive orders support US national plans for AI, including advancing leadership and harnessing national security, safety, and trust (The White House, 2023). Nevertheless, data solutions that develop enterprises with plans are not adequately equipped to reform the PHM of the HPO as a national strategy (Admin, 2024).

The UK Government’s DSIT global strategy scales safer and more responsible AI plans and actions (UK Government, 2021), but is not yet prepared for PHM reform through governed classification. Long-term NHSE plans for genomics and social models require ecosystem HPO-enabled predictors and intercept classifiers to benefit sectors and regions (Chapman and Middleton, 2019; Care planning, 2024). DSIT healthcare, scientific research, and digital transformation for the PHM of HPO use biological models for well-being with data-driven plans for proactive care (GOV.UK and DSIT, 2024). The HEMSS classifies valid pre-eXams and eXams to facilitate multicomplex model integration and simplify it using agile methodologies.

The UK-US PHM reform integrates HPO in joint NIST/AISI biological [Gen AI-X] models with national digital QA in neighborhood clinics or federal centers, which define innovation and culture pre-eXam/eXam classifiers for the Brightest Tomorrow (Kosiol et al., 2024; NIST, 2024). Figure 2 illustrates a conceptual PHM ecosystem proposed with HEMSS for deploying HPO-driven solutions that enhance healthcare delivery and improve patient outcomes; under national government authorities and directorship. This HEMSS initiative, envisioned as an executive arm of a corporate agile group development program, would aim to ensure the ethical use of the genome predictor pre-eXam for eXams intercepts, as personalized classifiers integrate the PHM ecosystem [X = Gen AI].

#### 1.3.3 Higher expert medical science safety agile group development

In Figure 1, the PHM is shown to transform primary care through HEMSS standards and tools. Agile groups have developed the NHSE Genomics and AISI evaluations for the adoption of AIDRS classifications for public health and patient safety. HEMSS aligns Healthcare Information Management System Society [HIMSS] norms in PHM governance in an ecosystem that adopts valid HPO (Healthcare Information and Management Systems Society, 2019). The UK AIDRS has developed NHSE HPO adoption in national clinical pathway agreements on fit-for-purpose pre-eXams/eXams across FDPs as HEMSS/HIMSS advances PHM maturity from 2025 (DHI Newsteam, 2022; Burrell, 2023).

In Figure 2, the upper circle informs the Human Phenotype Ontology Policy for UK Clinics and US Centers, which provides oversight of Value-Based Care at the point of need, while the smaller circle is under the authority of AIDRS and HEMSS. Figure 2 depicts the HEMSS HPO-centeredness with Gen AI = X for PHM deployment in genomic health pre-eXams and biopharma eXam intercepts, where HEMSS tools and principles encircle health providers and partners from left to right.

Table 1 illustrates the HEMSS with Agile Group Development, in which the PHM adopts HPO risk stratification for disease segmentation. Establishing a pilot in a UK ICB region does not preclude expansion, with the success of a pilot facilitating similar initiatives in the US that integrate HPO biological systems from neighborhood clinics to federal centers (NIST, 2024). Cross-national collaboration enhances PHM using advanced HPO technologies and ensures consistent improvements across both countries in a MoU on AI for PHM (UK.GOV, 2024).

**Table 1:** Higher expert medical science safety agile group development.

**HEMSS principles** *Requirements and interchangeable arrangements*	**Population health management of human phenotype ontology**
**Higher** **Infrastructure** *Classification for governance*	Hybrid servers provide crucial platforms for statistical-based predictive analytics and precision care with scalable HPO management for all
**Expert** **Agile development** *Governance of classification*	Agile group methods, like Sonnet 3.5 with future tooling, for adaptation to pre-eXam and eXam phase ideals provide evidence-based insight.
**Medical** **Practitioner and biopharma** *Engagement on classification*	Points of need established in agreements across practitioner and biopharma outlets are pioneered in UK clinics and US center services.
**Science** **Multi-omics–social factor** *Inclusiveness for classification*	Integrating scientific themes from multimodal data alliances upstream informatics like genomics, images, and socio-environmental factors.
**Safety** **Public health, safety, parity** *governance, and adherence*	Focus on public health, patient safety, and parity for an HPO primary care from personal healthcare responsibilities to government welfare.

Higher expert medical science safety (Underlined) refers to the conceptual ecosystem and agile group development initiative, designed to integrate principal stakeholders (Bold) for the ethical and effective management of human phenotype ontology within population health through classifications in national predictive health and precision care.

#### 1.3.4 HPO depicts predictor pre-eXams and precise care eXams classification

In Figure 1, the three towers are shown to develop agile GEN AI with the adoption of HPO primary care reform as an ecosystem that extends beyond the standard vocabulary. The author showcases pre-eXams and eXams using Gen-AI to predict human phenotypic abnormalities and target ontology intercepts related to standard traits or characteristics, with digital recommendations for optimal interventions. This manuscript assesses the actions and discusses the aims of biological modelling with socio-environmental elements for HPO ecosystem.

As shown in Figure 2, an HPO genome blueprint predicts and intercepts pathology in digital records that align pre-eXams with lifetime eXams for effective healthcare based on patient profiles. The flow of accurate eXams from HPO pre-eXam classifiers tailors the effective biopharma and socio-environmental intercepts. The post-eXam data refines the nodes with backpropagation for continual improvements. X alerts adoption, whereas X pre-exams/exams are not authorized for commission. An X could be invalid for multiple reasons, such as poor data training, unverified AI-QA, or excessive biopharmaceutical costs.

#### 1.3.5 Ontology system engineering initiatives for public safety

In Figure 2, in the lower circle, HPO System Engineering Initiatives for Public Safety (OSEIPS) is a proposal that aligns Population Health Management Higher Medical Science Safety Agile Group Development as the program proposal for the NHSE, Genomics England and the Life Science Sector to realize safe spaces. System Engineering Initiatives for Patient Safety (SEIPS) v 3.0 and SEIPS v 2.0 improve outcomes by applying principles from human factors and system thinking and understanding the interaction of the environment, tools, tasks, and people (Carayon et al., 2020; Holden et al., 2013).

In Figure 2, the bullet points across four healthcare sectors are detailed that impact patient safety, whereby the UK National Patient Safety Policy for NHSE recommends using SEIPS to learn from patient safety events (NHS England, 2019). The Patient Safety Incident Response Framework incorporates SEIPS to analyze and improve outcomes while Learning from Patient Safety Events (NHS England, n.d.a; NHS England, n.d.b). The Healthcare Safety Investigation Branch uses SEIPS as a monitoring tool to ensure comprehensive investigations and system-based improvements (GOV.UK, 2022).

#### 1.3.6 Assessment of well-being and welfare

**Figure 3:**
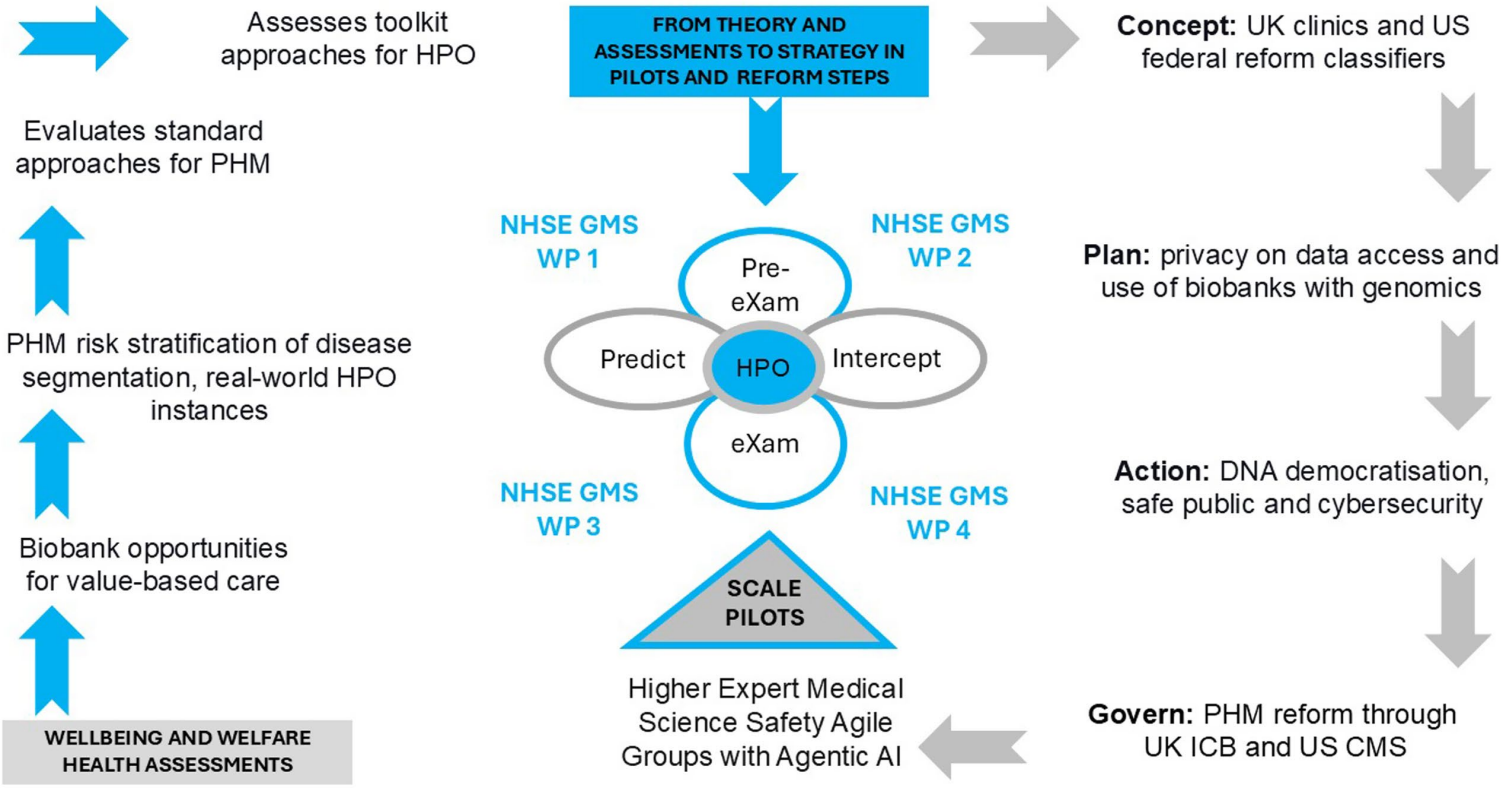
Population health management, health evaluation, reform steps and pilot that build on genomics towards HPO.

Figure 2 depicts the UK-US PHM AI aim for value-based care at the point of need across ICBs and CMS with HEMSS agile group development of HPO risk stratification and disease segmentation. The left-hand side of Figure 3 shows the well-being and welfare assessments encapsulating the PHM, with each step explained as follows. Section 1.3.7 details biobank opportunities for value-based care. Section 1.3.8 outlines PHM risk stratification of disease segmentation and real-world HPO instances. Section 1.3.9 evaluates the standard approaches for PHM, and Section 1.3.10 assesses the toolkit approaches for HPO.

#### 1.3.7 Biobank opportunities for value-based care

The UK-US PHM national genome studies with socioenvironmental factors have advanced HPO value-based care (HM Government, 2020; National Institutes of Health, 2019). UK PHM could solve medical challenges through programs such as the “Generation Study,” which sequences newborn genomes to identify rare genetic diseases, or the “Our Future Health” program, which collects health and lifestyle data for disease prevention and treatment of major conditions (Horton et al., 2024; Our Future Health and NHS, 2022). In the US, the “Million Veterans Program” collects genetic and health data to study how genes affect health, whilst the “All of Us” program gathers data and reaches out to partners to predict HPO and intercept pathology for well-being (Gaziano et al., 2016; NIH, 2024).

PHM biobank value-based care requires a normal toolkit approach to challenge opportune genome analysis, which classifies real-world HPO solutions (Oxford Academic Press, 2024). Literacy in predictive health biobanks from biological samples also models precision medicine, while the future direction classifies HPO predictors and intercepts for governance (Annaratone et al., 2021; Coppola et al., 2019). Biobanking now addresses complex predictive health and precision care in settings with evidence-based, real-world risk stratification and disease segmentation standards and tools to implement the national PHM successfully using the HEMSS task force (Vaught, 2021; Ede et al., 2024).

UK ICB assessments and US Centers for Medicare and Medicaid Services surveys for HPO developments require digital directories and modelling (N. England, 2023; CMS.GOV, 2023b; NIST, 2024). In this regard, clinical pathway initiatives for HPO systems such as familial hypercholesterolemia are supported by academia, science, and medicine, along with genome education on predictors and intercepts (Genomics Education, 2024; Chora and Bourbon, 2021). National AI evaluations of biological models and AIDRS project developments benefit from digital record pre-eXam and eXam classifications (NIST, 2024; The AI and digital regulations service, n.d.). The AIDRS authorities in research [HRA], regulation [MHRA], commission [NICE], and governance [CQC] may:

- Conduct a thorough review of all rare HPO diseases and major pathologies in the ecosystem.
- Develop classical genome predictors with intercepts and subsequently approve their adoption.
- Assign the classification of pre-eXam or eXam agile methodology as fit-for-purpose [X = Valid Gen AI].
- Align each X as a version of the predictor or intercept, which details the requirement set and arrangement met as explanans within the adopted X (The AI and digital regulations service, n.d.).

For well-being and welfare assessment, the proposition is that AIDRS authorities recommend agile group developers with X approval for adoption. The HEMSS provides public inclusivity and stakeholder engagement for developers to access data and for adopters to govern classification adherence. HEMSS further evaluates and acts on biobank opportunities for well-being and welfare value-based care by sustaining the digital PHM grid with:

1. HPO Policy development in the standard format, as nationally authorized.
2. Detailed principles for inclusiveness, engagement, governance, and adherence.
3. Classification of trusted research for evidence-based PHM, as approved by experts.
4. Ecosystem ontology system engineering for public safety, overseen by HEMSS stewards.

#### 1.3.8 PHM risk stratification of disease segmentation with real-world HPO instances

PHM disease segmentation is a healthcare tool that aligns intercepts and resources of common health conditions, such as agile grouping (Wood et al., 2023). However, the need for alignment in risk stratification necessitates demonstrating how disordered agile groupings can be practically applied (Wood et al., 2023). Cohorts present barriers to stratification and segmentation reforms, while valid HPO classifications improve public health outcomes and reduce adverse patient events (Chigboh et al., 2024). Biological models are built for integration with HPO, utilizing predictive health multi-omics pre-eXams to enable digital eXam intercepts as a reflex Gen AI response (NIST, 2024). Table 2 expands the real-world instances of predictive pre-eXam and eXam intercepts, detailing HPO activities.

**Table 2:** Real-world predictive pre-eXam and eXam intercept opportunities.

**pre-eXam**	**eXam**
Assessment of biological models	**Pre-eXam Concept** Predictive analysis of genetic data to identify disease or optimize therapy, like mono/poly gene risk scores or pharmacogenomics **The eXam Concept** Precision care with tailored multi-omics intercepts that span functional genomics from gene therapy, oligonucleotide, or protein
Analysis of HPO biological images	**Pre-eXam Concept** CNN whole-body MRI to a cell nucleolus scan instance masses to a gene translocation to predict or diagnose **The eXam Concept** CNN-based analysis of signals like ECG, digital blood cell morphology, and viscoelastic data is used to guide targeted anticoagulant and procoagulant therapies
Evaluation of biopharma tools	**Pre-eXam Concept** Predictive analysis of multi-omics use NLP in science literature to support credible biopharma foresight **The eXam Concept** Precise care through biomarker identification, drug discovery, and tailored treatments model HPO patterns to feature an intercept
Assessment of pathology	**Pre-eXam Concept** Virtual pathology could engage in simulating disease prediction, like sonnet 3.5 questionnaires, rare diseases, or major conditions. **The eXam Concept** Accurate intercepts are contrasted against traditional medical non-personalized approaches, like statins in CVD, for training
Analysis of primary care	**Pre-eXam Concept** Predictive health when scaled up would enable an immediate referral and an appropriate increase in correct referrals to a specialist **The eXam Concept** Precise care when scaled out would enable greater public access to servers that engage the pharmacist directly
Evaluation of social factors	**Pre-eXam Concept** Screening social factors in neighborhood clinics is useful to evaluate stress or behavior from low income or isolated patients **The eXam Concept** Knowledge of the social context may target individual or community intercepts to maximize benefit in neighborhood schemes
Assessment of mental health	**Pre-eXam Concept** Predictive health from a newborn genomics screen may indicate one of multiple health disorders that would present in adolescence **The eXam Concept** Precise care pre-arranges an appropriate psychologist specialist to assess a disorder, personalize treatment, and arrange support
Analysis of the environment	**Pre-eXam Concept** Environmental factors such as air quality, exposure to toxins, or access to green spaces are foreseen in geo-space data aggregates **The eXam Concept** Extends to ICB, local authorities, or organizations to implement control like reduced traffic flow, water purification, or gym facilities

The simplicity of PHM risk stratification of disease segmentation and its application to real-world HPO instances should keep our practitioners and readers engaged in the bigger picture of reform. Figure 1 depicts the HEMSS governance of classifications, adherence to predictor and intercept adoption, engaging stakeholders, and ensuring public inclusivity. These principles address broader socio-economic challenges. Figure 2 shows how PHM opportunities for HPO enhance quality of life and reduce premature deaths through contributions to the pre-eXams and eXams phases, ultimately mitigating community and global environmental risks through personalized plans for well-being and welfare. Figure 3 illustrates and reminds our society that the path to implementing reform follows steps including welfare and well-being evaluations of standard approaches for PHM, with an assessment of toolkit approaches for HPO.

#### 1.3.9 An evaluation of standard approaches for PHM

Quality data standards input heterogeneity for HPO output which interoperate with AI to predict and intercept pathology through FDPs in a safe space with a panoramic view of health for personalized plans (DHI Newsteam, 2022; Miandoab et al., 2023). Mature standards for interoperability provide advanced PHM opportunities.

#### 1.3.10 Maturity standards

Figure 2 shows the agile group developers who securely share and manage health information for the PHM of the HPO. The HIMSS Infrastructure Adoption Model (IFRAM) measures healthcare maturity, guiding organizations to optimize technology investments and improve outcomes (Healthcare Information and Management Systems Society, 2019; HIMSS, 2024b). The AMRAM evaluates the maturity of healthcare organizations in their use of analytics (HIMMS, 2021). The e-medical Record Adoption Model (EMRAM) guides the adoption of EHRs aimed at digital maturity and improved patient care (HIMSS, 2024a). HIMSS-EMRAM level 7 has robust analytics capabilities and strong data governance and uses technology for operational performance and best outcomes (Celebrating HIMSS stage 7 organizations advancing Global Health, 2020).

#### 1.3.11 Current interoperability standards

The International Classification of Diseases 11th revision is the global standard developed by the World Health Organization (WHO) for coding and classifying diseases, such as ORPHANET for rare disorders and related problems, to enhance diagnostic accuracy and support interoperability with modern digital systems (World Health Organization, 2022; GOV.UK, 2021). The ICD-10 is an earlier version that is widely used for coding and classifying medical diagnoses and mortality data to provide a common language for recording and reporting diseases (GOV.UK, 2021). HL7-FHIR is the norm for electronically exchanging healthcare information, promoting interoperability and seamless data sharing across ecosystems to improve healthcare delivery (HL7UK, “FHIR®,” HL7 UK, 2023).

#### 1.3.12 Advanced opportunity

Figure 1, Column 3 depicts HEMSS standards and tools for PHM predictive health pre-eXams and precise care eXams for each HPO. Biological modelling supports HPO understanding to advance ICD into safe HPO space (NIST, 2024) for dimensional points of need (Institute of Biomedical Sciences, 2023). HIMSS Level 7 ensures robust analytics capabilities and strong data governance, facilitating the use of advanced AI tools for improved patient outcomes (Celebrating HIMSS stage 7 organizations advancing Global Health, 2020). HEMSS agile groups further advance opportunities for the PHM of HPO classification stewardship in reforms on issues from rare infant diseases to non-communicable conditions with predictor pre-eXams and intercept eXams as the Gen X norm (GOV.UK, 2021; HL7UK, “FHIR®,” HL7 UK, 2023; GOV.UK, 2023b).

#### 1.3.13 An assessment of toolkit approaches for HPO

Figure 1 shows the PHM reform with the AI toolkit evaluated by the AISI for the AIDRS, executed by the HEMSS agile groups as a task force. Figure 3 depicts the PHM transformation from “theory and assessments to strategy in pilots and reform steps” using algorithms. AI Toolkit evaluations include Section 1.3.14, Adversarial, neural, and transformer, Section 1.3.15, Generative AI and multimodal data, and Section 1.3.16, Fair Artificial General Intelligence from unbiased Gen AI.

#### 1.3.14 Adversarial, neural and transformer tools

Figure 1 Column 1 highlights the UK-US infrastructure for PHM with AISI evaluation and AIDRS. Column 2 classifies VBC at ICB and CMS points of need. Column 3 shows AI toolkits for HPO systems that predict health (pre-eXams) for precision care (eXams). Comprehensive Neural Architecture Searches (NAS) and clusters deploy PHM solutions with HPO reinforcement for predictors and intercepts (Zoph and Le, 2017), as backpropagation recalibrates HPO systems in post-eXams. This approach must prioritize and optimize advanced AI infrastructure while transferring and centralizing techniques, e.g., imaging, which enhance PHM with effective and efficient HPO visions (Barret et al., 2017). Integrating NAS and patterning algorithms with evolutionary computation methods enhances PHM for HPO, examining the ongoing optimization of personalized plans with AI toolkits (Song et al., 2024).

In the left column of Table 3, pangenome Convolutional Neural Networks (CNNs) and Large Language Models (LLMs) address physical and mental health by analyzing layers and patterns in HPO systems to detect predictors and intercepts (NHSE, 2023). In the right column of Table 3, the benefits of Generative Adversarial Network (GAN) biopharma-biomarker development for HPO system personalized plans (NHSE, 2023) are enhanced by greater pan-genome specificity (Liao et al., 2023).

**Table 3:** Adversarial, neural, and transformer tools for HPO systems.

**Pre-eXam pangenome CNN and LLM**	**Generative adversarial network as eXams**
Learning sequence motifs	Generating artificial genome representation
Variant calls and classification	Data augmentation to improve performance
Ontology disorder prediction	Drug discovery
Ontology biopharma precision	Biomarker identification

Regarding well-being, GANs reform genomics by employing a generator and discriminator that compete with one another, measuring uncertainty, and optimizing HPO systems (Lee and Seok, 2021; Kim and Lee, 2023; Lee and Seok, 2019). These efforts have yielded landmark findings regarding gene expression (Lee, 2023). The GAN genome builds sustained features for PHM, reiterating complex patterns such as haplotypic structure and linkage disequilibrium (McVean and Kelleher, 2019). Pangenome specificity with GANs enhances the scientific aspects of nucleotide building, translating transcript machinery to proteins, advancing drug discovery, targeting binding affinity, and drug-to-drug interactions (Koutroumpa et al., 2023; Gangwal and Lavecchia, 2024; Kalemati et al., 2024; Tang et al., 2024), including epigenetic reflex testing, such as methylation (Sabrin Afroz et al., 2024).

We can picture CNNs in pre-eXam presentations of HPO nucleotide layers for motifs such as promoters, transcription factors, and epigenetic markers (Koo and Eddy, 2019). Concurrently, our researchers undertake extensive biological imaging for evidence-based routine services, which empowers personalized medicine with eXam intercepts and monitoring (Koo and Eddy, 2019; Galić et al., 2023). Indeed, the future of advanced medical pathology imaging provides the best patient benefits by backpropagating massive national outcome data sets (Sarvamangala and Kulkarni, 2021; Wang, 2024).

Regarding welfare, we transgress social science factors and environmental elements. LLMs are ideal for defining each HPO system within the biomedical and clinical domains (Yang et al., 2023), expanding a biological model with socio-environmental phenomena (NIST, 2024; Ziems et al., 2023). HPO systems align LLM data with HPO bioinformatics, biological models, and the social sciences (Ziems et al., 2023; Park et al., 2024; NIST, 2024). The vast increase in scientific LLM literature supports the aim to unravel PHM further (Authors, n.d.; Ding et al., 2023). Indeed, a future perspective of PHM assesses a biological model with clinical knowledge and social sciences using quality data and continuous integration for fair HPO (Manning et al., 2024; Singhal et al., 2023; Gallegos et al., 2023).

#### 1.3.15 Generative AI and multimodal data

Reform is a toolkit of promises in need of ecosystem thinking, wherein Generative AI improves organizational PHM capability to innovate and realize HPO risk stratification and condition segmentation (Wachter and Brynjolfsson, 2023). AI empowers health and social care to optimize performance and improve communication, providing novel insights into enhancing public health decision-making and outcomes (Bharel et al., 2024). The integration of Gen-AI in healthcare streamlines HPO flow for diagnostic accuracy and personalized treatment plans, advancing patient care and operational efficiency (Reddy, 2024). Gen AI models like transformers or diffusion models are reshaping predictive health and precision care. The eXams, digitally twinned from pre-eXam insights and alert to drug design or therapeutic targeting, may be guided by HPO imaging (Shokrollahi et al., 2023).

In Table 4, the PHM Gen AI multimodal data in genomics, images, and patient records feature HPO predictors for accurate intercepts. In Table 4, value-based care at points of need build genome biology models as socioenvironmental elements differentiate each HPO as a system.

**Table 4:** Generative AI and multimodal data.

**Population health management ecosystem** **points 1–6**	
**1 Genome**	**2 Biological model**
Identify variants associated with diseases	Develop AI tools to analyze NGS and omics
Personal treatment plans from a genome	Computational ontology to simulate biology
Predict drug response/side effect by gene	Analyze genome-protein interaction to target drug
Design drug targeting to genetic mutation	Metabolome and disease market discovery
Novel genes associated with complex traits	Transcriptome and disease subtyping
Gene-editing therapies for gene disorders	Proteome and drug target prediction
Synthetic genomes to model human diseases	Epigenetics and disease market discovery

**3 Human phenotype ontology**	**4 HPO pathology differential causation**
Evaluate NAS to reason over ontology data	LLM extracts social factor text information
Evaluate data to create knowledge	Knowledge graph representation between entities
Evaluate errors in existing ontologies	Identify factor patterns in ontology data
Automate creations and curation of ontology	Integrate ontology to EHR interoperability
Transform ontology with rare disease predictor	Ontology image reports with social factor reviews
Transform ontology with chronic disease assessment	Chronic disease management from determinants
Transform ontology using personal drug therapy	Health equity intervention on social diversities

**5 Human phenotype ontology as a system**	**6 Population health management ecosystem**
Transform ontology with a single visual scan	Ontology image reports with social factor reviews
Align physical condition risk assessments	Oncology image reports with social factor reviews
Mental health assessment support	Community health surveillance
Personalized drug therapies	Health equity intervention on social diversities

In Figure 2, the HPO is centered on Gen AI and realized across sectors using synthetic or real-world multimodal data, wherein ecosystem analytics for GANs, CNNs, and LLMs are operational (Bragazzi and Garbarino, 2024). NHS Genomic England governs the “blueprint” that characterizes insights, initiating risk stratification for predictors while segmenting conditions for intercepts with value-based care at the point of need for truth (Institute of Biomedical Sciences, 2023).

#### 1.3.16 Fair artificial general intelligence from unbiased gen AI

Figure 3 shows the steps to PHM for HPO, while quantum intelligence will execute the WHO-UN promise to improve well-being by mitigating patient risks for socioeconomic growth (United Nations, 2023; U. Environment, 2021). A balance of what is fair in public health and welfare edges Gen AI within Q-Star and Sonnet 3.5 projecting biological modelling toward digital HPO expertise (Indian Express, 2023; NIST, 2024). PHM with quantum ML advances complex data handling through fault tolerance to sustain health goals (Wang and Liu, 2024; United Nations, 2023). Agentic AI involves continuous HPO monitoring in a post eXam phase which also mitigate bias, as explained below.

1. Gen-AI GANs in HPO biology may introduce data bias during training and mislead genomics, transcriptomics, and pathology imaging (Lacan et al., 2023). Properly trained GANs enhance HPO well-being in an ecosystem of diverse welfare (Cai et al., 2024; Gomathi et al., 2024). Robust GANs must demonstrate fairness in well-being and welfare evaluations.
2. Gen-AI CNNs in pathology may introduce biases if image analysis negates gene data or disease segmentation, which requires national biological model training (Kshatri and Singh, 2023; Kourounis et al., 2023). Graphs of humans in their environments assist in risk stratification and identifying social factor predictors of HPO disease for well-being and welfare intercepts (Zhang et al., 2019; Molokwu et al., 2020; Obeidat et al., 2023).
3. Gen AI LLMs for HPO analyze scientific data with trust in the actions identifying candidate gene selection and roles of dark data in disease (Birhane et al., 2023; Toufiq et al., 2023). LLMs of social factors provide a mindset of a non-clinical disposition to phenotypic points of need, with each bias noted and adjusted for a fair predictor and intercept (Patra et al., 2021; Mass General Brigham, 2024).

Meanwhile, the fairness of quantum Agentic AI in national access and the use of personal data must determine the moral intentions for sustaining people in decent work if we are truly to sustain well-being and welfare (UK Parliament, 2024; United Nations, 2023; U. Environment, 2021).

#### 1.3.17 The population health management of ontology, a strategy for reform

In the realm of well-being, medical errors have been identified as the third leading cause of death, with the epidemiology of malpractice claims significantly impacting health and socioeconomic growth (Makary and Daniel, 2016; Wallace et al., 2013). While it is human to err, our practitioners adhere to the “first do no harm” principle in building a safer health and care ecosystem (Kohn et al., 2000).

In Table 5, population health management of human phenotype ontologies as a strategy for UK healthcare reform lays the foundation for theories to construct ecosystem excellence for public health, patient safety, and parity (NHSE, 2024). The UK Department of Science, Innovation, and Technology are designing a robust infrastructure with key recommendations for science and technology to reform populace health (GOV.UK and DSIT, 2024).

**Table 5:** Theory on building a PHM reform strategy for public health, patient safety, and parity.

**Digital transformation**	**AI integration**	**PHM improvement**
Cybernetic	Social	General
Information	Personality	Open
Mathematics	Behavior	Living
Value	Culture	World

Figure 3’s right-hand side illustrates the PHM strategy as a series of detailed reform steps, which are explained as follows. Section 1.3.18 conceptualizes UK clinics and US federal reform classifiers. Section 1.3.19 outlines the planning of privacy for data access and use of genomic biobanks. Section 1.3.20 details the action plan for DNA democratization, safe public health, and cybersecurity. Section 1.3.21 discusses governing boards and services in a PHM reform and Section 1.3.22 outlines the scaling of higher expert medical science safety with agile groups.

#### 1.3.18 Conceptualizing UK clinics and US federal reform classifiers

In Figure 3, the first step to reform involves conceptualizing UK clinics and US federal reform with populace health classifiers that deploy fit-for-purpose analytics as primary HPO healthcare. Fully understanding primary care reform is an international effort to address heterogeneity of aging in biological medicine and to classify genome predictive and diagnostic health in the pre-eXam (Henderson et al., 2023). PHM resources streamline processes, improve transparency, eliminate duplication, and mitigate variations in practice to accelerate concise and accurate eXam intercepts and reduce health disparities for outcome excellence in a lifetime (Wise, 2024). Table 6 aligns UK-US clinic services with a global focus on new PHM themes that realize HPO value-based care at points of need.

**Table 6:** Comparison of UK ICBs and US CMSs that integrate reform.

**UK integrated** **care board**	**Themes**	**US center for medicaid and medicare services**
Neighborhood Clinics, Community Diagnostic Centers	Primary care facilities	Community Health Centers and Federally Qualified Health Centers
Integrated Primary Care (IPC) and public health services for access equity	Focus	IPC and public health services for low-income and uninsured populations
NHS England, ICBs, and local authorities	Governance	Federal criteria – Department of Health and Human Resources
Funded by the UK Government over 42 regions	Funding	Federal criteria and receives funding under the Public Health Service Act
IPC addressing both medical and social needs with community AI support	Service model	IPC and preventive services serving underserved areas and populations
Stakeholder engagement channels for inclusivity on reform	Community involvement	Governed by a community board, with members being patients of the clinic
National insurance contribution and free at the point of use	Patient fees	Services provided on a sliding fee scale based on income
Resource and integration transforming with a new agile infrastructure	Structure challenges	Addressing health in a fragmented insurance system
Population Health Management of Human Phenotype Ontology will accelerate in the UK	New Global Focus	Population Health Management of Human Phenotype Ontology will benefit the US from a MoU on PHM
HEMSS pilot programs with Agile Group Development improve outcomes on effective points of need	Pilot Digital Programs	HEMSS pilot programs with Agile Group Development lower costs and improve value-based care
Predictive health pre-eXams, precision care eXams, and AISI Biological modelling with governance	Value-based Care - Points of Need	Predictive health pre-eXams, precision care eXams, and AISI Biological modelling with governance

A new global focus on digital platforms, pilot programs, and value based care at points of need for truth complements genomics and life sciences through the adoption of patient-centered approaches. It also supports the objectives of 10-year plans in health and social care by improving efficiency, equity, and responsiveness as an ecosystem.

#### 1.3.19 Planning privacy on data access and use of biobanks with genomics

The US CMS provides extensive data access through Data.CMS.gov, which collects and shares data on Medicare and Medicaid beneficiaries, highlighting steady enrolment increases of healthcare providers and citizen service users (Data.CMS.Gov, n.d.). Other HPO data under the “CMS Innovation Centre Programs” appears fragmented (Data.CMS.Gov, n.d.). Multiple datasets improve patient care and reduce costs, with CMS developers accessing biobanks and primary care data for the PHM of HPO through the Million Veterans and All of Us Programs sourced with genomics and socio-environmental informatics (Gaziano et al., 2016; NIH, 2024; CMS Developer, 2024).

The Health Insurance Portability and Accountability Act privacy rule ensures that patient data are protected and used appropriately, as provided in the summary of the privacy rule (U.S. Department of Health and Human Services, 2022). Although the benefits of PHM are evident, they must operate within HIPAA’s strict data privacy and security standards to protect patient data and use them appropriately with the components of a model representative of HPO (Team DataMotion, 2019). HIPAA sets national standards for protecting Protected Health Information (PHI), ensuring that data privacy and security are maintained while allowing the flow of health information needed for high-quality care (OCR, 2008). HIPAA regulations require detailed attention, with checklists available for comprehension and adherence to new data security measures that may affect biobank data use (HIPAA Journal, 2022).

The UK Integrated Care Ecosystem and ICBs are responsible for protecting the privacy of identifiable information and ensuring that data sharing between healthcare providers complies with the Data Protection Act 2018 and the UK GDPR (GOV.UK, 2018). Developers must implement robust data protection measures, such as encryption and access controls, to safeguard patient information and ensure data is handled securely and transparently within a PHM ecosystem; failure to comply with these regulations could result in corrective actions, sanctions, and penalties (Information Commissioner's Office, 2023). Biobank agile groups in the UK must adhere to standards that protect their HPO data, while adopters of PHM must be aware of the impact of new legislation.

1. The Data Protection and Digital Information Bill focuses on regulating the processing of personal data, privacy, and e-communications while strengthening the Information Commission to enhance data security, with UK data rights for scheduled PHM through consent of what an individual wishes to know (GOV.UK, 2023a).
2. The Data (Use and Access) bill is designed to unlock the secure and effective use of data for public interest by including interoperability and data sharing across health and social care sectors, ensuring accurate and secure data access, which impacts our HPO (UK Parliament, 2024).

In Figure 3, the steps for PHM use of UK-US biobanks are shown to prioritize privacy for individuals, who should understand and agree on how personal data are used for HPO well-being. HIPAA requires covered entities to obtain patient consent for the use and disclosure of PHI while mandating authorization for the use or disclosure of PHI not otherwise permitted by the Privacy Rule, ensuring that patients are informed about how their data will be used and shared (U.S. Department of Health and Human Services, 2022; Team DataMotion, 2019; OCR, 2008; HIPAA Journal, 2022). The UK Data (Use and Access) Bill enhances data governance and transparency, with provisions for informed consent and explicit authorization from individuals to use their data involving AI and digital processes such as PHM, which would be required (GOV.UK, 2023a).

The Acts and Bills with which authorities aim to address privacy with AI in decision-making while ensuring transparency and fairness support the development of a data ecosystem that promotes well-being and drives socioeconomic growth (U.S. Department of Health and Human Services, 2022; Team DataMotion, 2019; OCR, 2008; HIPAA Journal, 2022; GOV.UK, 2023a; UK Parliament, 2024). The PHM’s potential to exchange data across international borders and maximize data aggregation to enhance equality and global health is most feasible with anonymized data and citizen-informed consent to support a worldwide HPO standard of care with federated Antigenic AI learning (Köhler et al., 2021).

#### 1.3.20 Action plan DNA democratization, safe public health, and cybersecurity

On new frontiers, science and technology reforms are accelerated by engaging boundaries for DNA use, providing evidence for PHM to predict and intercept HPO with government cybersecurity guardrails (Schumacher et al., 2020; GOV.UK and DSIT, 2024). Genomic tests for disease must be cyber-secure, as we strive for DNA passports and quality lives that maintain good health and data protection while negating vulnerability (Bilkey et al., 2019; Mayeur et al., 2023). The UK and US MoU for AI in PHM aims for safe science and secure technology to ensure global health and individual privacy (UK.GOV, 2024). Citizen mistrust or perceptions of transparency may lead individuals to refuse genome access, although DNA democratization with or without passports ensures an identification legitimacy within a safe well-being space (Mayeur et al., 2023; Tommel et al., 2023), which HEMSS strives for.

As shown in Figure 1, standard data AI tools with agile groups develop HPO in cybersecure classifications that protect against threats while being transparent and unbiased. The National Science and Technology Governance of PHM disease segmentation for nucleotide-HPO intercepts introduced another X dimension for privacy, security, and health parity (Department for Science, Innovation & Technology, 2024). The joint AISI pre-deployment biological models evaluate the safety and security needs for cyber-secure PHM ecosystems, as Gen AI aligns biological questions with answers (NIST, 2024). Safe public health in a cyber-secure nation involves test evaluation cycling through vulnerability, discovery, and exploitation to ensure robust operations, agile ecosystem environments, and seamless attack planning and execution (NIST, 2024).

#### 1.3.21 Stewarding PHM reform through UK ICB and US CMS with HEMSS

In the UK, audits in the healthcare sector and community assessments contribute to a more extensive set of data alliances, evaluating informatics for quality improvement (Healthcare Quality Improvement Partnership, 2024; NHS England, 2019; NHS England, 2017; UK Health Data Research, 2024). Genomic data are a promising aspect of the future of NHSE assessment of ICBs, with accountability in evaluating PHM performance to improve well-being as a national strategic priority (N. England, 2023; Genome UK, 2022). The UK Rare Diseases and Major Conditions unit’s prediction and intercept arrangements (GOV.UK, 2021; GOV.UK, 2023b) benefit profoundly from infant studies and adult programs that build on biological modelling (NHS England, 2024; Our Future Health, 2021). The truth is that UK organizations are not prepared for the PHM of the HPO, so the HEMSS is necessary to ready reform in a UK-US MoU (Figure 1).

Oversight in HPO reform uses a new global pan-genome reference to accelerate genomic specificity and enrich value-based care (National Human Genome Research Institute, 2023; NHS England, 2022). The UK government’s direction in neighborhood clinic reform is to direct resources to primary care, which benefits from the HEMSS principles (Anderson, 2024) (Figure 1, Column 2). Meanwhile, the NHSE infrastructure with QA governance developed a digital genomic directory service for HPO predictors and intercept services as an agile group development (NHSE, 2024; C. Office, 2023). Meanwhile, value-based care in the UK-US MoU aligns social determinants with genomics, benefiting the US Center for Medicare and Medicaid Innovation (Holland and Knight, 2024; Office of Disease Prevention and Health Promotion, 2023), as both projects deliver on the Biological Model (NIST, 2024).

The US Department of Health and Human Services supports structured knowledge as CMS surveys aim to mitigate premature deaths and unnecessary emergency department arrivals (Authors, 2023; Centers for Disease Control and Prevention, 2019). Risk factor surveillance is misplaced in disease control, while PHM for HPO in a HEMSS toolkit benefits CMS federal centers with value-based care (Figure 1). Federal Center PHM of the HPO model will implement value-based primary care by incorporating risk scores and pharmacogenomics as pre-eXam model queries, wherein the X will detail the particulars of the query as the point of truth or probability (Lewis et al., 2024; CMS.GOV, 2023a; Collister et al., 2022; Sarwar, 2023). With over 1 million registrants in the “All of Us” program a national PHM reform has commenced (All of Us Research Hub, 2024). Therefore, HEMSS principles and classifications for the UK-US joint aim to model HPO (NIST, 2024) as reform classifiers to initiate controlled change (Figure 1).

#### 1.3.22 Scaling higher expert medical science safety with agile groups

UK and US healthcare services face increasing demand, complexity, costs, and competencies that benefit PHM economically by scaling science and technology (Spanos, 2024; GOV.UK and DSIT, 2024). AI’s advantage, combined with genomics’ big data veracity and velocity and the variety and volume of health determinants, presents HPO opportunities to enhance PHM (Keskar et al., 2020). The UK Infant Generation Study and digital records deepen biological models, augmenting phenotypic risk stratification and pathology segmentation and presenting an international opportunity (Horton et al., 2024).

The International Genome Health pre-eXam and our future life in science infrastructure, with authority and directorship (Figure 1), guide HPO policy projects using HEMSS agile groups (Figure 2) and fit-for-purpose reform steps (Figure 3). The PHM of rare infant diseases extends to non-communicable condition predictors with practical and ethical intercepts as the population health projects reform services in diagnostics and conventional medicine (Population Health Analytics Laboratory, 2024). In medical specialty analytics such as hematology and cardiovascular disease, HEMSS agility and stewardship pilot Gen AI proof-of-concept with biological commissioning scaling population health excellence (The Royal College of Pathologists, 2020). Consider the following depictions:

1. In Figure 3, the PHM HEMSS Agile Group reform steps ensure the capability and capacity for AI methods across health to build pilot projects. One project depicted a pre-eXam-eXam proof-of-concept in a community of one infant disorder for a digital genome test to confirm a sickle cell variant in the classification of a CRISPR eXam (C. Office, 2023). Pilots inform the analytics, trials, and authorities of transparent points of need, while X is through development in research to commission for adoption through an AIDRS (NICE) authority (NHS Beta, n.d.).
2. As shown in Figure 2, HIMSS-IFRAM engineers adopt Gen AI-X classifications to level seven EMRAM assessments in the built-from-test directories to personalized biological models in a safe space (Healthcare Information and Management Systems Society, 2019; HIMSS, 2024b; HIMMS, 2021; HIMSS, 2024a; Celebrating HIMSS stage 7 organizations advancing Global Health, 2020; NIST, 2024). Storage is required for the data of 100,000 infants and upscaled computing power is needed for WGS VCF capacity in neighborhood clinics (NHS England, 2024). Capacities for multiethnic communities and pathological disorders are generalized for pediatricians and midwives, with AI training of workflows provided with policy, provider, and community stakeholders, as depicted in Figure 2, points.
3. In Figure 1, the HEMSS Agile Group biological model principles (Column 2) with norms and tools (Column 3) execute the HPO in the next generation of primary care. Scaling out PHM use and access requires public inclusivity, stakeholder engagement in governance, adherence to predictors, and intercept classification. AIDRS - HEMSS steward the PHM of HPO under NHSE, Genomics England and Life Science directorship. HEMSS Agentic AI align pre-eXam and steward eXams for well-being within digital twin classifications.

The pathological pilots align with other WGS predictors to provide a differential diagnosis between biological and HPO models. The PHM of HPO commission and adopt excellence in national multi-omics, imaging and social determinants for primary stakeholders in the Genomics England, the Royal Colleges and bio-banks as the AI laboratory services develop with the proposed HEMSS stewardship which align ecosystem digital approaches (Population Health Analytics Laboratory, 2024; The Royal College of Pathologists, 2020; Royal College of Pathologists, 2022).

### 1.4 The UK and US memorandum of understanding for PHM

In 2021, the US infant mortality rate was 5.4 deaths per 1,000 live births, while the UK rate was 3.7 (CDC, 2024; Office for National Statistics, 2023). The UK also has a higher average life expectancy (ONS, 2024; Centers for Disease Control and Prevention, 2023). These statistics highlight the need for continued efforts to transform public health outcomes with the PHM of predictors and intercepts in safe spaces impacted by digital genomics test directories and biological modelling as public health primary care (Khoury and Holt, 2021; Scott et al., 2019). By 2030, the UK ICBs and US CMSs aim for semantically interoperable HPO decision support (de Mello et al., 2022; Jing et al., 2022), which benefits from HEMSS stewardship of ICBs and CMS across health and social care sectors.

In the UK-US MoU for AI-PHM, the proposal for HEMSS Agile Groups engages our society, as illustrated in Figure 1 and detailed in Table 1, with the required principles, standards, and tools that integrate HPO on behalf of the respective national authorities. Section 1.4.1 debates HPO as a form of ethical and societal primary care, while Section 1.4.2 discusses HEMSS for global public health inclusiveness and national engagement. Section 1.4.3 discusses HEMSS to steward the classification phases for adherence, while Section 1.4.4 debates PHM for UK and US accreditation or regulations.

#### 1.4.1 Debating HPO as an ethical and societal primary care

From a UK perspective, NHSE and Genomics England actively incorporates PHM as a central strategy across ICBs to transform a reactive healthcare system into a proactive ecosystem, addressing inequalities and optimizing outcomes (NHS England, 2021b). The delay in providing patient data to biobanks is due to the need for a federated learning ecosystem for PHM processing, where the choice of critique may cite ethical, social, and regulatory prerequisites (Rumbold and Pierscionek, 2017). Nevertheless, practitioners and the public must consider the moral and societal positive implications of PHM to determine the HPO (Yurkovich et al., 2023; Hiam et al., 2024). Table 7 presents the ethical debates on integrating HPO reform into primary care.

**Table 7:** Ethical debates on integrating HPO.

**Topics of interest that occur** **for the PHM of HPO**	**Ethical debates** **for and against**
Multi-omics and health determinant EHRs model HPO, which benefits from a personalized Digital Cloud record to personalize plans. As NHSE GMS integrate classifiers, they educate on HPO benefits. HEMSS coordinates inclusiveness for individuals to trust national ID DNA while educating people on the major benefits over the minor risks of genomic data integration.	**For**: Enhance personalized treatment plans and predictive health access. Builds trust through public education with the inclusiveness of families. **Against**: Raises concerns about data privacy and security with a potential misuse of genomic data.
Stakeholders would engage in “Population Health Management Higher Expert Medical Science Safety with Agile Development,” with biobank - technologies for the PHM of HPO in versions over a lifetime. It would be standard to engage AI toolkit and Information Technology security for data access and safe decision-making, as VBC is provided at the point of need.	**For**: Promotes innovation and better decision-making. HIMSS/HEMSS ensures agile data accuracy and robust security. Both technology and science advance in a unified vision. **Against**: Potential for unequal access to advanced technologies. Risk of data breaches.
Biobanks serve stakeholders for UK and US clinics and centers for primary care pre-eXams and biopharma or life choice eXams phases through public IDs that intercept accurate healthcare with data aggregation. Predictors and diagnosis use analytics to intercept HPO biological and social factors. PHM risk stratifies with Gen AI when user consent has been agreed.	**For**: Improves public health and personalized care. Enhances disease prediction and prevention while mitigating premature death. **Against**: Ethical concerns about data accuracy and patient consent. Potential for discrimination based on genetic data.
OSEIPS manages the behavior and physical HPO phase processes for control with HEMSS governance of classifications that facilitate directorship of primary care reform. HPO pre-eXams transform in clinics and sectors use HIMSS and ISO 15189:2022 norms for AI (GAN, CNN, LLM, and Gen AI) safe space governance, assurance, and training of multimodal data.	**For**: Ensures responsible deployment of AI in healthcare. Continually improves healthcare services. Outputs provide public confidence. **Against**: This may create bureaucratic hurdles and slow innovation with a potential for over-reliance on AI.
Regulation, accreditation policies, norms, and tools for HPO reform with Cloud biobanks agile phase classifications stratify risks and segment disease for accurate intercepts. AIDRS authority and NHSE NEQAS- GM’s directorships with AGI for PHM of HPO reach out for data aggregation of medical, scientific, and biopharma data for OSEIPS solutions to improve public health, patient safety, and parity	**For**: Improves public health, patient safety, and parity. Supports digital regulation and AI safety. Aligns ecosystem order for PHM **Against**: Risk of widening existing health disparities. Potential for regulatory overreach. Resistance from bodies or leaders to reform.

As Figure 3 shows, implementing reform steps in PHM for the HPO has societal implications involving bias, patient autonomy, and public engagement in decision-making regarding AI predictors and intercepts. Table 8 discusses the societal impacts of the AI PHM toolkit for public HPO, inclusivity, and stakeholder engagement while governing the classifications for adherence to HEMSS principles.

**Table 8:** Societal debates on integrating HPO.

**Societal** **topics**	**Ecosystem argument** **for the PHM of HPO**	**Arguments against PHM of HPO with HEMSS principles**
Bias in AI algorithms	AI helps to identify or mitigate existing bias in risk stratification to predict and then intercept disease segmentation	Algorithms perpetuate and amplify biases if not QA, leading to inaccurate stratification or pathology segmentation [HEMSS engagement is national QA]
	Properly designed AI can enhance fairness and equity in healthcare delivery, particularly in the PHM of HPO through newborn sequencing	Biased AI can lead to unfair treatment with health disparities, undermining the goals of PHM for HPO. [HEMSS governs valid and verified classifications]
Impact on patient autonomy	AI empowers people with information and personalized treatment options, supporting better risk stratification and pathology segmentation	AI reliance undermines autonomy and doctor-patient bonds. [HEMSS inclusivity and engagement aligns the public with stakeholders]
	AI tools support public health and shared decision-making between patients and healthcare providers for effective PHM of HPO.	Patients may feel choice is limited by AI, reducing PHM take up [HEMSS classify multiple citizen options in QA eXams with probability scores on the best choices]
Involvement in decision-making	Involving the public in AI decision-making ensures transparency and trust, which is required for effective PHM for HPO	Public engagement processes can be time-consuming and may slow PHM for HPO [HEMSS classify X evidence for informed choices]
	Public input can lead to more socially acceptable and ethical AI applications, improving the overall impact of PHM for HPO	People may misunderstand or resist safe QA-AI technologies, which hinder PHM for HPO [HEMSS refusal of classification options provide structured review]

#### 1.4.2 HEMSS global public health inclusiveness and national engagement

In Table 1, HEMSS principles support global attitudes and behaviors for PHM as NHSE and Genomics England plans digital test directories for universal points of need (NHS England, 2021b; Institute of Biomedical Sciences, 2023; C. Office, 2023). Indeed, as a prelude to PHM, the WHO evaluated the Healthy City Program and highlighted its impact on urban health and policies (de Leeuw et al., 2015). Many PHM studies have developed themes for trust in the vision of shared ownership and goals, with place-based accountable transformation to an ecosystem (Siegel et al., 2018; Pimperl, 2018; Greater Manchester Integrated Care Partnership, n.d.), whereas the HEMSS stewardship systemizes HPO for global health inclusiveness.

The WHO Focus Group for evaluating standard AI health solutions interfaces ML, medicine, regulation, public health, and ethics (United Nations, n.d.) with HEMSS principals, engaging stakeholders on reform to UN SDGs (United Nations, 2023; U. Environment, 2021). Meanwhile, a PHM ecosystem with newer pangenomes determines HPO specificity, providing safer predictors and intercepts (Petrić Howe and Bundell, 2023). Moving with the WHO’s six principles, the direction intensifies on the genome and social science in public health inclusiveness with national engagement to optimize the PHM of HPO through HEMSS future directions (Jasarevic, 2021; Benjamin et al., 2024).

Furthermore, the WHO cautions against using AI in poorer nations, speculating that the UK-US should lead global initiatives, whereby the HEMSS is ideal for the PHM of HPO reform with evidence-based outcomes (Adam, 2024). Looking through the lens of heterogeneity, HPO are associated with scientific features that predict health and inform intercepts through a classical approach (Woodward et al., 2022). UK NHSE AI Ops, Genomics England, and the US NIH alignment for AISI/NIST open biological models, while HEMSS simplifies science and technology reform with a “classical” progress unique to national HPO integration (Department of Science, Innovation and Technology, 2023; Human Phenotype Ontology, n.d.).

Figure 1 depicts the development of GEN AI for adoption in primary care reform. In Column 2, HEMSS principles deploy value-based care through public inclusiveness and stakeholder engagement in the governance and adherence to fit-for-purpose classifications at the point of need. In Column 3, the HEMSS standards and tools for the UK ICB and US CMS execute the classical predictive health pre-eXams and eXam intercepts, with each X explaining world HPO health and what nations choose to adopt for their PHM development.

#### 1.4.3 HEMSS stewards classification phases for adherence and harmonization

Figure 3 harmonizes the reform steps and pilots involving US-UK partners in AI Safety focused on biological modelling for personalized predictors and intercepts (NIST, 2024). The MoU for AI establishes the AISI for the future of HPO, with pilot projects through the AIDRS with Genomics England, biobanks, Google, DeepMind, and OpenAI (US Department of Commerce, 2024; NHS Beta, n.d.; Care planning, 2024). HEMSS stewards the classifier phases in personalized predictive health pre-eXams and precise care eXam intercepts.

Figure 2 harmonizes value-based care points of need for HPO-centered healthcare that is safe, fair, transparent, trustworthy, and engages organizations overseen by UK ICBs and the US CMS underpinned by national health initiatives. PHM develops from Trusted Research Environments, with UK NIHR and US NIH engaging in clinical pathway initiatives, using specialist genomics to develop ecosystem PHM of HPO with Agentic AI stewards of classifications (National Institute for Health and Care Research, 2024; National Institute of Health, 2024b).

Figure 1 harmonizes the reform columns for the PHM-HEMSS agile group development, featuring multiple authorities that align into more refined services with authority for HPO research, regulation, commission, and stewardship underpinned by Generative AI in quantum futures. Table 9 shows the changing landscape, as the management of populace health will realign national services that harmonize with digital identity.

**Table 9:** PHM harmonization aligns national organizations for HPO.

**UK population** **health management**	**US population** **health management**
**Human Pangenome Reference Consortium**	**Human Pangenome Reference Consortium**
*The International Human Pangenome sets a genetic baseline, crucial for public inclusiveness in the development of HPO digital identities for PHM.*	
**Genomics Medical Services**	**National Human Genome Research Institute**
*Engagement between GMS and health research institutes identifies predictors in multi-omics data, essential for personalized care plans during the digital lifetime.*	
**National Institute of Health Research**	**Centers for Disease Prevention and Control**
*Nations align on health risk stratification research, enabling better disease segmentation with accurate intercepts aligned using the digital record.*	
**NHS AI Laboratory [Biobanks]**	**National Institute of Health**
*QA-tailored analytics for personalized care in safe and secure digital spaces for ICB and CMS oversight of digital maturity HIMSS and HEMSS for PHM of HPO reform.*	
**Responsible Technology Adoption**	**National Institute of Standards & Technology**
*Trust in technology adoption, accept statistical outcomes, and mitigate adverse events, like premature death or reduced life expectancy, through adherence to digital classifications.*	
**Technical PRIDAR**	**Clinical PRIM&R**
*Engaging stakeholders in AI aims to ensure the integrity of HPO systems. HEMSS agile group development mitigates risks in the algorithm and clinical service.*	
**AI Digital Regulation Service**	**Food and Drug Administration**
*The ecosystem expands data alliances service adherence as scientific themes develop classifications for adoption by organizations.*	
**AI Safety Institute**	**AI Safety Institute**
*The UK-US AISI for PHM aims to predict and intercept HPO, with ecosystem ICB and CMS organizational adherence to HIMSS-HEMSS infrastructure.*	

#### 1.4.4 Debate on future PHM for US-UK accreditation or regulation

As Figure 2 shows, the HPO policy stewards the predictive health pre-eXam development and precise care eXam adoption in an ecosystem of fit-for-purpose classification adherence and monitoring. Roadmaps for digital transformation in the UK and US align with HPO service reviews to consider PHM regulations or accreditation with access and the reuse of data for value-based care at a point of need (Transformation Directorate, n.d.; National Institutes of Health, n.d.). The debates follow the themes regarding accreditation and regulation in Table 10, and a discussion summary follows.

**Table 10:** The PHM of HPO for accreditation or regulation.

Human phenotype ontology accreditation	Themes	Population health management regulation
**For**: Ensures quality and standards. **Against**: What is to be accredited?	Quality	**For**: Ensures compliance and safety. **Against**: Multi-stakeholder complexity
**For**: Builds trust and credibility. **Against**: Costly and time-consuming	Cost	**For**: Increases public confidence. **Against**: Expensive to implement
**For**: Provides continuous improvement. **Against**: May limit innovation.	Innovation	**For**: Ongoing enhancements. **Against**: Resistance to reform requires HEMSS stewardship.
**For**: Transparency and accountability. **Against**: Create bureaucratic hurdles	Inclusivity	**For**: Transparency and accountability. **Against**: Lead to excessive red tape.
**For**: Facilitates international recognition. **Against**: Not universally accepted.	Adherence	**For**: Ensures global standards! **Against**: Different national laws
**For**: Stakeholder engagement. **Against**: Can be seen as restrictive	Engagement	**For**: Stakeholder involvement **Against**: May limit flexibility

## 2 Discussion summary

In Figure 1 Column 2, US accreditation of HPO VBC or UK PHM regulation of points of need are shown to be complementary approaches that review national structures and governance. US HPO accreditation means deciding what to accredit while implementing comprehensive ecosystems with data access, a federal priority for digital predictive health pre-eXam and precise eXam intercepts as classifications (CMS.GOV, 2024; NCQA, 2018; The Joint Commission, 2024). There is global momentum to advance HPO quality from research using standard vocabulary in PHM Agentic AI for classification, stewardship, and adherence (Gargano et al., 2023).

In Figure 2, the small circles A contain UK and US HEMSS stewards for agile groups in PHM, stewarding the predictors and intercepts. Regulating digital PHM with safe algorithms in a robust ecosystem develops points of need to adopt HPO truth in the pre-eXams and eXams with privacy and cybersecurity (US Department of Commerce, 2024; NHS Beta, n.d.). The PHM trains on data aggregated for GenAI, where HPO X is a fit-for-purpose pre-eXam and eXam in personalized plans with lifecycle versions. Pre-exam/exam with a de-sized x, informed predictor, or intercept is not recommended for adoption, citing the reason from the AIDRS in explaining x.

In Figure 3, for the best outcome, the federated learning quantum intelligence reform steps engage PHM stakeholders to accept or reject HPO flow as a predictor or intercept that is X-approved for adoption, which mutes the accreditation vs. regulation debates for continual improvements. HEMSS-Agentic AI reduces bureaucratic hurdles to ecosystem quality and safety, while ethical queries on accountability, transparency, human autonomy, privacy, bias, and job security remain (Botha et al., 2024; Masters et al., 2024; Sun et al., 2024). The PHM HPO governor, accreditor, or regulator of quantum Agentic AI classifies predictors and intercepts for adherence to value-based care at the point of need with truth or probability (NHS England, 2021b; Institute of Biomedical Sciences, 2023), which is ethical and suitable for public health, patient safety, and parity.

## 3 Conclusion

The international program proposal for “Population Health Management Higher Expert Medical Science Safety (HEMSS) Agile Group Development” resonates with global healthcare through its reform impact. HEMSS people inclusivity and stakeholder engagement improve public health, patient well-being, and parity at the point of need for truth or probability. The PHM classification develops a fit-for-purpose HPO for point-of-need adoption with value-based predictors and intercepts in an ecosystem.

A UK and US MoU for AI and global aims for HPO by 2030 organizes for world health and sustained development goals with HEMSS a reform complement to HIMSS assents for PHM adherence. The UK and US PHM strategy and HPO policy for agile development monitors public safety in biological and social determining evaluations. Assessments of well-being and welfare utilize biobanks with genomics as HEMSS norms and AI tools accelerate the global aims for PHM of HPO.

The author tabulates the reform and HEMSS governance with agile group development (Table 1) and classifies instances of real-world predictive pre-eXam and eXam intercepts (Table 2). The program uses adversarial, neural, and transformer tools for HPO systems (Table 3) with generative AI and multimodal data (Table 4), as society adopts theories to develop infrastructure (Table 5).

To transform HPO theory into PHM practice, HEMSS agile groups were developed with ICBs and CMSs (Table 6). Ethical and societal debates have ensued on integrating HPO (Tables 7, 8). The PHM actions align national services with debates on accreditation and regulation for the future of Agentic AI (Tables 9, 10). The author’s work builds on his original concept, later expedited by NHSE, for quality assurance of end to end workflow including validation, training, and assurance (Henry, 2014, unpublished manuscript)^1^. This foundational idea now informs the development of HEMSS agile groups, which accelerate public health, patient safety, and equality through science and technology governed by classification adherence as appropriated to ISO 15189:2022 Annex A (Royal College of Pathologists, 2022).

The UK and US national structures and policies for PHM benefit from a strategy toward biological modelling and data monitoring, as expedited in predictive health pre-eXams and precise eXam intercepts [X = HPO-Gen AI]. UK clinics continue to progress with their genome working phase strategy as biobanks expand their social data. Concurrently, more effort is needed in the US States for seamless value-based care across citizens’ HPO points of need. The value of HEMSS agile group development is in clear principal policies, standards, tools, and HPO monitoring, which accelerate PHM reform through classic digital identities.

The National Health Service Act of 1946 and US Medicare and Medicaid Act of 1965 benefit from safe and secure HPO systems. The stewarding proposition for national oversight executes public inclusivity, stakeholder engagement, governance of classifications, and adherence to principles, standards, and tools for global reform to develop PHM.

Recognising new organisational strategies with HEMSS through bodies, such as the UK Genomics England and the Life Science Sector is profound in realising the NHS 10 Year plan, “Population Health Management, Higher Expert Medical Science Safety with Agile Group Development” is a proposal to the World Health Organization to sustain development goals for well-being and socioeconomic growth. In conclusion, international oversight should consider the principles of the HEMSS stewardship for public wellbeing with ecosystem value-based care in population health at the point of phenotype need for the truth in medicine.

## Footnotes

^1^J. A. Henry, "Patient Blood Management," unpublished manuscript, supervised by NHS England Workflow Governance Lead, University of Greenwich, Greenwich, U.K., 2014.

## Glossary

AGI: Artificial General Intelligence
AIDRS: AI Digital Regulation Service
AISI: AI Safety Institute
CMS: Centers for Medicare and Medicaid Services
CNN: Convoluted Neural Networks
CQC: Care Quality Commission
CVD: Cardiovascular Disease
DNA: Deoxy Ribonucleic Acid
DRS: Digital Regulation Service
DSIT: Department of Science, Innovation, and Technology
ECG: Electrocardiograph
EHR: Electronic Health Record
EMRAM: E-Medical Record Adoption Model
FDP: Federated Data Platform
FHIR: Fast Healthcare Interoperability Resources
GAN: Generative Adversarial Network
GDPR: General Data Protection Regulation
GEN: AI Generative AI
GMS: Genomic Medical Service
GP: General Practitioner
GPU: General Processing Units
HEMSS: Higher Expert Medical Science Safety
HIMSS: Healthcare Information Management System Society
HIPAA: Health Insurance Portability and Accountability Act
HPO: Human Phenotype Ontology
HRA: Health Research Authority
ICB: Integrated Care Board
ICD: International Classification of Diseases
IFRAM: Infrastructure as a Model
IPC: Integrated Primary Care
ISO: International Organization of Standardization
LLM: Large Language Models
MHRA: Medicines and Healthcare products Regulatory Agency
ML: Machine Learning
MoU: Memorandum of Understanding
MRI: Magnetic Resonance Imaging
NAS: Neural Architecture Search
NEQAS: National External Quality Assurance Service
NHS: National Health Service
NHSE: NHS England
NIST: National Institute of Standards and Technology
NICE: National Institute of Clinical Excellence
NLP: Natural Language Processing
NSCT: National Science and Technology Council
OSEIPS: Ontology System Engineering Initiatives for Public Safety
PCHR: Personalized Cloud Health Records
PHI: Protected Health Information
PHM: Population Health Management
PRIDAR: Predictive Risk Intelligence Decision Analytics and Reporting
QA: Quality Assurance
SEIPS: System Engineering Initiatives for Patient Safety
TRE: Trusted Research Environment
VBC: Value-Based Care
WHO: World Health Organization

## References

[ref1] Adam D. (2024). Medical AI could be ‘dangerous’ for poorer nations, WHO warns. Nature. doi: 10.1038/d41586-024-00161-1, PMID: 38243121

[ref2] Admin. (2024). How can advanced data solutions transform healthcare delivery. smartData, Available online at: https://www.smartdatainc.com/knowledge-hub/how-can-advanced-data-solutions-transform-healthcare-delivery/ (Accessed December 09, 2024)

[ref3] Alderwick H. Hutchings A. Mays N. (2024). Cross-sector collaboration to reduce health inequalities: a qualitative study of local collaboration between health care, social services, and other sectors under health system reforms in England. BMC Public Health 24, 1–19. doi: 10.1186/s12889-024-20089-5, PMID: 39334058 PMC11438096

[ref4] All of Us Research Hub. (2024). Data snapshots – All of us research hub. Available online at: https://researchallofus.org/data-tools/data-snapshots/ (Accessed August 24, 2024)

[ref5] Anderson M.. (2024). Labour party manifesto: ‘shifting resources to primary care’ and a ‘National Care Service,’ Available online at: https://www.nursinginpractice.com/latest-news/labour-party-manifesto-shifting-resources-to-primary-care-and-founding-a-national-care-service/ (Accessed August 24, 2024).

[ref6] Annaratone L. de Palma G. Bonizzi G. Sapino A. Botti G. Berrino E.. (2021). Basic principles of biobanking: from biological samples to precision medicine for patients. Virchows Arch. 479, 233–246. doi: 10.1007/s00428-021-03151-0, PMID: 34255145 PMC8275637

[ref8] Authors. (2023). Centers for Medicare & Medicaid Services, CMS’ value-based programs. CMS. Available online at: https://www.cms.gov/medicare/quality/value-based-programs (Accessed August 23, 2024).

[ref9] Authors. (n.d.) Mapping the increasing use of LLMs in scientific papers. Available online at: https://arxiv.org/html/2404.01268v1 (Accessed May 16, 2024)

[ref10] Barret Z. Vasudevan V. K. Shlens J. Le Q. V. (2017). “Learning transferable architectures for scalable image recognition,” arXiv. (Cornell University). doi: 10.48550/arxiv.1707.07012

[ref11] Benjamin D. Cesarini D. Turley P. Strudwick Young A. (2024). Social-science genomics: Progress, challenges, and future directions. Natl. Bureau Econ. Res. doi: 10.3386/w32404

[ref12] Bharel M. Auerbach J. Nguyen V. DeSalvo K. B. (2024). Transforming public health practice with generative artificial intelligence. Health Aff. 43, 776–782. doi: 10.1377/hlthaff.2024.00050, PMID: 38830160

[ref13] Bilkey G. A. Burns B. L. Coles E. P. Bowman F. L. Beilby J. P. Pachter N. S.. (2019). Genomic testing for human health and disease across the life cycle: applications and ethical, legal, and social challenges. Front. Public Health 7:40. doi: 10.3389/fpubh.2019.00040, PMID: 30915323 PMC6421958

[ref14] Birhane A. Kasirzadeh A. Leslie D. Wachter S. (2023). Science in the age of large language models. Nat. Rev. Physics 5, 277–280. doi: 10.1038/s42254-023-00581-4

[ref15] Botha N. N. Segbedzi C. E. Dumahasi V. K. Maneen S. Kodom R. V. Tsedze I. S.. (2024). Artificial intelligence in healthcare: a scoping review of perceived threats to patient rights and safety. Arch. Public Health 82:188. doi: 10.1186/s13690-024-01414-1, PMID: 39444019 PMC11515716

[ref7] Bragazzi N. L. Garbarino S. (2024). Toward clinical generative AI: conceptual framework. JMIR Preprints. Available at: https://preprints.jmir.org/preprint/55957 (Accessed August 4, 2025). 10.2196/55957 PMC11193080 38875592

[ref16] Brown E. A. White B. M. Jones W. J. Gebregziabher M. Simpson K. N. (2021). Measuring the impact of the affordable care act Medicaid expansion on access to primary care using an interrupted time series approach. Health Research Policy Systems 19:77. doi: 10.1186/s12961-021-00730-0, PMID: 33957934 PMC8101185

[ref17] Burrell D. (2023). The path to HIMSS level 5 for the NHS. BridgeHead Software. Available online at: https://www.bridgeheadsoftware.com/2023/02/the-path-to-himss-level-5-for-the-nhs/ (Accessed May 13, 2024).

[ref18] C. Office. (2023). National genomic test Directory_2023-2025 - UK NEQAS. Available online at: https://ukneqas.org.uk/news/nhs-england-genomic-medicine-service/national-genomic-test-directory_2023-2025/ (Accessed August 24, 2024).

[ref19] Cai Z. Xiong Z. Xu H. Wang P. Li W. Pan Y. (2024). Generative adversarial networks. ACM Comput. Surv. 54, 1–38. doi: 10.1145/3459992, PMID: 39076787

[ref20] Carayon P. Wooldridge A. Hoonakker P. Hundt A. S. Kelly M. M. (2020). SEIPS 3.0: human-centered design of the patient journey for patient safety. Appl. Ergon. 84:103033. doi: 10.1016/j.apergo.2019.103033, PMID: 31987516 PMC7152782

[ref21] Care planning. (2024). Describe the biomedical, social and ecological models of health and well-being - care learning. Care Learning, Available online at: https://carelearning.org.uk/qualifications/level-3-extended-hsc/hsc-cm7/1-4-describe-the-biomedical-social-and-ecological-models-of-health-and-well-being/ (Accessed December 09, 2024)

[ref22] CDC. (2024). Infant mortality. Maternal Infant Health. Available online at: https://www.cdc.gov/maternal-infant-health/infant-mortality/index.html (Accessed August 27, 2024).

[ref23] Center for AI and Digital Policy. ABOUT, center for AI and digital policy. Available online at: https://www.caidp.org/about-2/ (Accessed December 09, 2024).

[ref24] Centers for Disease Control and Prevention. (2019). CDC – BRFSS. Available online at: https://www.cdc.gov/brfss/index.html (Accessed August 29, 2024).

[ref25] Centers for Disease Control and Prevention. (2023). Life expectancy. Available online at: https://www.cdc.gov/nchs/fastats/life-expectancy.htm (Accessed August 27, 2024).

[ref26] Chapman R. Middleton J. (2019). The NHS long term plan and public health. BMJ 364:l218. doi: 10.1136/bmj.l218, PMID: 30651224

[ref27] Chigboh V. M. Christophe Zouo S. J. Olamijuwon J. (2024). Health data analytics for population health management: a review of best practices and challenges. Int. J. Front. Med. Surgery Res. 6, 106–116. doi: 10.53294/ijfmsr.2024.6.2.0050

[ref28] Chora J. R. Bourbon M. (2021). Pharmacogenomics of statins and familial hypercholesterolemia. Curr. Opin. Lipidol. 32, 96–102. doi: 10.1097/mol.0000000000000746, PMID: 33591029

[ref29] CMS Developer. (2024). Data. Available online at: https://developer.cms.gov/data-cms/ (Accessed December 14, 2024)

[ref30] CMS.GOV. (2023b). Resuming validation of accrediting organization surveys | CMS. Available online at: https://www.cms.gov/medicare/provider-enrollment-and-certification/surveycertificationgeninfo/administrative/resuming-validation-accrediting-organization-surveys (Accessed August 27, 2024)

[ref31] CMS.GOV. (2023a). Making care primary (MCP) model | CMS. Available online at: https://www.cms.gov/priorities/innovation/innovation-models/making-care-primary (Accessed August 24, 2024).

[ref32] CMS.GOV. (2024). Search | CMS. Available online at: https://www.cms.gov/search/cms?keys=accreditation (Accessed December 16, 2024).

[ref33] Collister J. A. Liu X. Clifton L. (2022). Calculating polygenic risk scores (PRS) in UK biobank: a practical guide for epidemiologists. Front. Genet. 13:818574. doi: 10.3389/fgene.2022.818574, PMID: 35251129 PMC8894758

[ref34] Coppola L. Cianflone A. Grimaldi A. M. Incoronato M. Bevilacqua P. Messina F.. (2019). Biobanking in health care: evolution and future directions. J. Transl. Med. 17:172. doi: 10.1186/s12967-019-1922-3, PMID: 31118074 PMC6532145

[ref35] Crane M. Nathan N. McKay H. Lee K. Wiggers J. Bauman A. (2022). Understanding the sustainment of population health programmes from a whole-of-system approach. Health Research Policy Systems 20:37. doi: 10.1186/s12961-022-00843-0, PMID: 35392913 PMC8988542

[ref36] Data.CMS.Gov. Available online at: https://data.cms.gov/ (Accessed December 14, 2024)

[ref37] de Leeuw G. Green M. Dyakova L. S. Palmer N. (2015). European healthy cities evaluation. Health Promot. Int. 30, i8–i17. doi: 10.2307/48519637, PMID: 26069320

[ref38] de Mello B. H. Rigo S. J. da Costa C. A. da Rosa Righi R. Donida B. Bez M. R.. (2022). Semantic interoperability in health records standards: a systematic literature review. Heal. Technol. 12, 255–272. doi: 10.1007/s12553-022-00639-w, PMID: 35103230 PMC8791650

[ref39] Department for Science, Innovation & Technology. (2024). UK screening guidance on synthetic nucleic acids for users and providers, Available online at: https://www.gov.uk/government/publications/uk-screening-guidance-on-synthetic-nucleic-acids/uk-screening-guidance-on-synthetic-nucleic-acids-for-users-and-providers (Accessed Dec. 14, 2024)

[ref40] Department of Science, Innovation and Technology, (2023). The UK science and technology framework taking a systems approach to UK science and technology. Available online at: https://assets.publishing.service.gov.uk/media/6405955ed3bf7f25f5948f99/uk-science-technology-framework.pdf (Accessed December 26, 2023)

[ref41] DHI Newsteam. (2022). The role of a successful federated data platform programme. Digital Health. Available online at: https://www.digitalhealth.net/2022/09/the-role-of-a-successful-federated-data-platform-programme/ (Accessed November 26, 2023)

[ref42] Ding Q. Ding D. Wang Y. Guan C. Ding B. (2023). Unraveling the landscape of large language models: a systematic review and future perspectives. J. Electron. Business Digital Econ. 3, 3–19. doi: 10.1108/jebde-08-2023-0015, PMID: 35579975

[ref43] Donohue J. M. Cole E. S. James C. V. Jarlenski M. Michener J. D. Roberts E. T. (2022). The US medicaid program: coverage, financing, reforms, and implications for health equity. JAMA J. Am. Med. Assoc. 328, 1085–1099. doi: 10.1001/jama.2022.14791, PMID: 36125468

[ref44] Ede A. F. T. M. Minderhout R. N. Stein K. V. Bruijnzeels M. (2024). How to successfully implement population health management: a scoping review. BMC Health Serv. Res. 23:910. doi: 10.1186/s12913-023-09915-5, PMID: 37626327 PMC10464069

[ref45] Galić I. Habijan M. Leventić H. Romić K. (2023). Machine learning empowering personalized medicine: a comprehensive review of medical image analysis methods. Electronics 12, –4411. doi: 10.3390/electronics12214411

[ref46] Gallegos I. O. Rossi R. A. Barrow J. Tanjim M. M. Kim S. Dernoncourt F.. (2023). Bias and fairness in large language models: A survey. doi: 10.48550/arXiv.2309.00770

[ref47] Gangwal A. Lavecchia A. (2024). Unlocking the potential of generative AI in drug discovery. Drug Discov. Today 29:103992. doi: 10.1016/j.drudis.2024.103992, PMID: 38663579

[ref48] Gargano M. Matentzoglu N. Coleman B. Addo-Lartey E. B. Anagnostopoulos A. V. Anderton J.. (2023). The human phenotype ontology in 2024: phenotypes around the world. Nucleic Acids Res. 52, D1333–D1346. doi: 10.1093/nar/gkad1005, PMID: 37953324 PMC10767975

[ref49] Gaziano J. M. Concato J. Brophy M. Fiore L. Pyarajan S. Breeling J.. (2016). Million veteran program: a mega-biobank to study genetic influences on health and disease. J. Clin. Epidemiol. 70, 214–223. doi: 10.1016/j.jclinepi.2015.09.016, PMID: 26441289

[ref50] Genome UK. (2022). Shared commitments for UK-wide implementation 2022 to 2025. Available online at: https://www.gov.uk/government/publications/genome-uk-shared-commitments-for-uk-wide-implementation-2022-to-2025/genome-uk-shared-commitments-for-uk-wide-implementation-2022-to-2025 (Accessed August 23, 2024)

[ref51] Genomics Education. (2024). The clinical pathway initiative - genomics education Programme. Genomics Education Programme. Available online at: https://www.genomicseducation.hee.nhs.uk/the-clinical-pathway-initiative/ (Accessed December 02, 2024)

[ref52] Gomathi B. Saravana Balaji B. Krishna Kumar V. Abouhawwash M. Aljahdali S. Masud M.. (2024). Multi-objective optimization of energy aware virtual machine placement in cloud data center. Intelligent Automat Soft Computing 33, 1771–1785. doi: 10.32604/iasc.2022.024052, PMID: 40612875

[ref53] GOV.UK. (2018). Data protection act. Available online at: https://www.gov.uk/data-protection (Accessed July 28, 2023)

[ref54] GOV.UK. (2021). UK rare diseases framework. Available online at: https://www.gov.uk/government/publications/uk-rare-diseases-framework (Accessed October 01, 2023).

[ref55] GOV.UK. (2022). Health and care bill: health services safety investigations body. Available online at: https://www.gov.uk/government/publications/health-and-care-bill-factsheets/health-and-care-bill-health-services-safety-investigations-body#:~:text=How%20These%20Provisions%20Help%20Improve%20Public%20Confidence (Accessed January 20, 2023)

[ref56] GOV.UK. (2023b). Major conditions strategy: case for change and our strategic framework. Available online at: https://www.gov.uk/government/publications/major-conditions-strategy-case-for-change-and-our-strategic-framework/major-conditions-strategy-case-for-change-and-our-strategic-framework--2 (Accessed October 01, 2023)

[ref57] GOV.UK. (2023a). Data protection and digital information bill: impact assessments. Available online at: https://www.gov.uk/government/publications/data-protection-and-digital-information-bill-impact-assessments (Accessed Aug. 13, 2023)

[ref58] GOV.UK and DSIT. (2024). Artificial Intelligence Sector Study. Available online at: https://assets.publishing.service.gov.uk/media/671a06ca67b3ef4856faf8a8/artifical_intelligence_sector_study_2023.pdf (Accessed December 09, 2024)

[ref59] Greater Manchester Integrated Care Partnership, Home. greater Manchester integrated care partnership. Available online at: https://gmintegratedcare.org.uk/ (Accessed December 16, 2024)

[ref60] Healthcare Information and Management Systems Society. (2019). Who we are | HIMSS. Available online at: https://www.himss.org/who-we-are (Accessed December 10, 2022).

[ref61] Healthcare Quality Improvement Partnership. (2024). Laying the FOUNDATION for improvement in healthcare. Available online at: https://www.hqip.org.uk/wp-content/uploads/2023/11/HQIP_Cornerstone_2024.pdf (Accessed August 23, 2024)

[ref62] Henderson D. Donaghy E. Dozier M. Guthrie B. Huang H. Pickersgill M.. (2023). Understanding primary care transformation and implications for ageing populations and health inequalities: a systematic scoping review of new models of primary health care in OECD countries and China. BMC Med. 21:319. doi: 10.1186/s12916-023-03033-z, PMID: 37620865 PMC10463288

[ref63] Hiam L. Klaber B. Sowemimo A. Marmot M. (2024). NHS and the whole of society must act on social determinants of health for a healthier future. BMJ 385:e079389. doi: 10.1136/bmj-2024-079389, PMID: 38604669

[ref64] HIMMS. (2021). Adoption model for analytics maturity (AMAM) | HIMSS. Available online at: https://www.himss.org/what-we-do-solutions/digital-health-transformation/maturity-models/adoption-model-analytics-maturity-amam#:~:text=Adoption%20Model%20for%20Analytics%20Maturity%20%28AMAM%29%20The%20HIMSS (Accessed December 10, 2022)

[ref65] HIMSS. (2020). Celebrating HIMSS stage 7 organizations advancing Global Health | HIMSS. Available online at: https://www.himss.org/news/celebrating-himss-stage-7-organizations-advancing-global-health (Accessed May 13, 2024).

[ref66] HIMSS. (2024a). Electronic medical record adoption model (EMRAM) | HIMSS. Available online at: https://www.himss.org/what-we-do-solutions/maturity-models-emram (Accessed May 13, 2024)

[ref67] HIMSS. (2024b). Infrastructure adoption model (INFRAM) | HIMSS. Available online at: https://www.himss.org/what-we-do-solutions/maturity-models-infram (Accessed May 13, 2024).

[ref68] HIPAA Journal. (2022). New HIPAA regulations in 2019. HIPAA Journal Available online at: https://www.hipaajournal.com/new-hipaa-regulations/ (Accessed December 14, 2024).

[ref69] HL7UK. (2023). Available online at: https://www.hl7.org.uk/standards/hl7-standards/fhir/ (Accessed December 18, 2024).

[ref70] HM Government. (2020). GENOME UK the future of healthcare. Available online at: https://assets.publishing.service.gov.uk/government/uploads/system/uploads/attachment_data/file/920378/Genome_UK_-_the_future_of_healthcare.pdf (Accessed: March 11, 2023)

[ref71] Holden R. J. Carayon P. Gurses A. P. Hoonakker P. Hundt A. S. Ozok A. A.. (2013). SEIPS 2.0: a human factors framework for studying and improving the work of healthcare professionals and patients. Ergonomics 56, 1669–1686. doi: 10.1080/00140139.2013.838643, PMID: 24088063 PMC3835697

[ref72] Holland and Knight. (2024). Key value-based care developments to watch in 2024. Insights. Holland & Knight. Available online at: https://www.hklaw.com/en/insights/publications/2024/03/key-value-based-care-developments-to-watch-in-2024 (Accessed August 23, 2024).

[ref73] Horton R. Wright C. F. Firth H. V. Turnbull C. Lachmann R. Houlston R. S.. (2024). Challenges of using whole genome sequencing in population newborn screening. BMJ 384:e077060. doi: 10.1136/bmj-2023-077060, PMID: 38443063

[ref74] Human Phenotype Ontology, Human phenotype ontology. Available online at: hpo.jax.org. https://hpo.jax.org/ (Accessed December 16, 2024).

[ref75] Indian Express. (2023). What is project Q*, the AI breakthrough from OpenAI? 5 reasons why it may threaten humanity. The Indian Express, Available online at: https://indianexpress.com/article/technology/artificial-intelligence/project-q-star-explained-openai-sam-altman-9041746/ (Accessed December 07, 2024).

[ref76] Information Commissioner's Office. (2023). Enforcement of this code. Available online at: https://ico.org.uk/for-organisations/uk-gdpr-guidance-and-resources/data-sharing/data-sharing-a-code-of-practice/enforcement-of-this-code/ (Accessed December 14, 2024).

[ref77] Infrastructure and Projects Authority. (2021). Transforming infrastructure performance: roadmap to 2030. Available online at: https://www.gov.uk/government/publications/transforming-infrastructure-performance-roadmap-to-2030/transforming-infrastructure-performance-roadmap-to-2030 (Accessed December 09, 2024)

[ref78] Institute of Biomedical Sciences. (2023). Point of care testing: National Strategic Guidance for at point of need testing. Institute of Biomedical Science. Available online at: https://www.ibms.org/resources/documents/point-of-care-testing-national-strategic-guidance-for-at-point/ (Accessed May 10, 2024).

[ref79] Jasarevic T.. (2021). WHO issues first global report on artificial intelligence (AI) in health and six guiding principles for its design and use. Available online at: https://www.who.int/news/item/28-06-2021-who-issues-first-global-report-on-ai-in-health-and-six-guiding-principles-for-its-design-and-use (Accessed May 28, 2024).

[ref80] Jing X. Min H. Gong Y. Sittig D. F. Biondich P. Robinson D.. (2022). A systematic review of ontology-based clinical decision support system rules: usage, management, and interoperability. medRxiv (Cold Spring Harbor Laboratory). doi: 10.1101/2022.05.11.22274984

[ref81] Kalemati M. Emani M. Z. Koohi S. (2024). DCGAN-DTA: predicting drug-target binding affinity with deep convolutional generative adversarial networks. BMC Genomics 25:411. doi: 10.1186/s12864-024-10326-x, PMID: 38724911 PMC11080241

[ref82] Keskar V. Yadav J. Kumar A. H. (2020). 5V’s of big data attributes and their relevance and importance across domains. Regular 9, 154–163. doi: 10.35940/ijitee.k7709.0991120

[ref83] Khatib H. S. A. Neupane S. Manchukonda H. K. Golilarz N. A. Mittal S. Amirlatifi A.. (2024). Patient-centric knowledge graphs: a survey of current methods, Challenges and Applications. doi: 10.48550/arXiv.2402.12608 PMC11558794 39540199

[ref84] Khoury M. J. Holt K. E. (2021). The impact of genomics on precision public health: beyond the pandemic. Genome Med. 13:67. doi: 10.1186/s13073-021-00886-y, PMID: 33892793 PMC8063188

[ref85] Kim J. Lee M. (2023). Portfolio optimization using predictive auxiliary classifier generative adversarial networks. Eng. Appl. Artif. Intell. 125:106739. doi: 10.1016/j.engappai.2023.106739

[ref86] Köhler S. Gargano M. Matentzoglu N. Carmody L. C. Lewis-Smith D. Vasilevsky N. A.. (2021). The human phenotype ontology in 2021. Nucleic Acids Res. 49, D1207–D1217. doi: 10.1093/nar/gkaa1043, PMID: 33264411 PMC7778952

[ref87] Kohn L. T. Corrigan J. M. Donaldson M. S., (2000). To err is human: building a safer health system. PubMed, Available online at: https://pubmed.ncbi.nlm.nih.gov/25077248/ (Accessed Dec. 18, 2024) 25077248

[ref88] Koo P. K. Eddy S. R. (2019). Representation learning of genomic sequence motifs with convolutional neural networks. PLoS Comput. Biol. 15:e1007560. doi: 10.1371/journal.pcbi.1007560, PMID: 31856220 PMC6941814

[ref89] Kosiol J. Silvester T. Cooper H. Alford S. Fraser L. (2024). Revolutionising health and social care: innovative solutions for a brighter tomorrow – a systematic review of the literature. BMC Health Serv. Res. 24:809. doi: 10.1186/s12913-024-11099-5, PMID: 38997711 PMC11241933

[ref90] Kourounis G. Elmahmudi A. Thomson B. Hunter J. Ugail H. Wilson C. (2023). Computer image analysis with artificial intelligence: a practical introduction to convolutional neural networks for medical professionals. Postgrad. Med. J. 99, 1287–1294. doi: 10.1093/postmj/qgad095, PMID: 37794609 PMC10658730

[ref91] Koutroumpa N.-M. Papavasileiou K. D. Papadiamantis A. G. Melagraki G. Afantitis A. (2023). A systematic review of deep learning methodologies used in the drug discovery process with emphasis on in vivo validation. Int. J. Mol. Sci. 24:6573. doi: 10.3390/ijms24076573, PMID: 37047543 PMC10095548

[ref92] Kshatri S. S. Singh D. (2023). Convolutional neural network in medical image analysis: a review. Archives Comput. Methods Engineering 30, 2793–2810. doi: 10.1007/s11831-023-09898-w

[ref93] Lacan A. Sebag M. Hanczar B. (2023). GAN-based data augmentation for transcriptomics: survey and comparative assessment. Bioinformatics 39, i111–i120. doi: 10.1093/bioinformatics/btad239, PMID: 37387181 PMC10311334

[ref94] Lee M. (2023). Recent advances in generative adversarial networks for gene expression data: a comprehensive review. Mathematics 11:3055. doi: 10.3390/math11143055

[ref95] Lee M. Seok J. (2019). Controllable generative adversarial network. IEEE Access 7, 28158–28169. doi: 10.1109/access.2019.2899108

[ref96] Lee M. Seok J. (2021). Estimation with uncertainty via conditional generative adversarial networks. Sensors 21:6194. doi: 10.3390/s21186194, PMID: 34577397 PMC8471214

[ref97] Legisltaion.Gov.UK. (1946). National health service act 1946. Available online at: https://www.legislation.gov.uk/ukpga/Geo6/9-10/81/enacted (Accessed December 07, 2024)

[ref98] Lewis C. Bryan A. Horstman C.. (2024) Federally qualified health centers can make the switch to value-based payment, but need assistance. Available online at: Commonwealthfund.org

[ref99] Liao W.-W. Asri M. Ebler J. Doerr D. Haukness M. Hickey G.. (2023). A draft human pangenome reference. Nature 617, 312–324. doi: 10.1038/s41586-023-05896-x, PMID: 37165242 PMC10172123

[ref100] Makary M. A. Daniel M. (2016). Medical error—the third leading cause of death in the US. BMJ 353:i2139. doi: 10.1136/bmj.i2139, PMID: 27143499

[ref101] Manning B. S. Zhu K. Horton J. J. (2024). Automated social science: language models as scientist and subjects. arXiv (Cornell University). doi: 10.48550/arxiv.2404.11794

[ref102] Mass General Brigham. (2024). Generative artificial intelligence models effectively highlight social determinants of health in doctors’ notes | mass general Brigham. Available online at: https://www.massgeneralbrigham.org/en/about/newsroom/articles/generative-artificial-intelligence-models-effectively-highlight-social-determinants-of-health-in-doctors-notes (Accessed May 25, 2024)

[ref103] Masters K. Herrmann-Werner A. Festl-Wietek T. Taylor D. (2024). Preparing for artificial general intelligence (AGI) in health professions education: AMEE guide no. 172. Med. Teach. 46, 1258–1271. doi: 10.1080/0142159x.2024.2387802, PMID: 39115700

[ref104] Mayeur C. Mertes H. Van Hoof W. (2023). Do genomic passports leave us more vulnerable or less vulnerable? Perspectives from an online citizen engagement. Human. Soc. Sci. Commun. 10:83. doi: 10.1057/s41599-023-01580-7, PMID: 36909259 PMC9985078

[ref105] McVean G. Kelleher J. (2019). Linkage disequilibrium, recombination and haplotype structure. Wiley online library, 51–86. doi: 10.1002/9781119487845.ch2

[ref107] Miandoab A. T. Samad-Soltani T. Jodati A. Rezaei-Hachesu P. (2023). Interoperability of heterogeneous health information systems: a systematic literature review. BMC Med. Inform. Decis. Mak. 23. doi: 10.1186/s12911-023-02115-5, PMID: 36694161 PMC9875417

[ref108] Molokwu B. C. Bhatta Shuvo S. Kar N. C. Kobti Z. (2020). Node classification in complex social graphs via knowledge-graph Embeddings and convolutional neural network. Lect. Notes Comput. Sci, 183–198. doi: 10.1007/978-3-030-50433-5_15

[ref109] N. England. (2023). NHS England» annual assessment of integrated care boards 2023/24 – supporting guidance. Available online at: https://www.england.nhs.uk/long-read/annual-assessment-of-integrated-care-boards-2023-24-supporting-guidance/ (Accessed August 30, 2024)

[ref110] National Archives. (2022). Medicare and Medicaid act (1965). National Archives. Available online at: https://www.archives.gov/milestone-documents/medicare-and-medicaid-act (Accessed December 07, 2024)

[ref111] National Committee for Quality Assurance in Collaboration with Health Management Associates, Population health management: Meeting the demand for value-based care National Committee for quality Assurance in Collaboration with health management associates. Available online at: https://www.ncqa.org/wp-content/uploads/2021/02/20210202_PHM_White_Paper.pdf (Accessed December 07, 2024)

[ref112] National Human Genome Research Institute. (2023). A new human ‘pangenome’ reference. Available online at: https://www.genome.gov/about-genomics/new-human-pangenome-reference (Accessed July 06, 2024)

[ref113] National Institute for Health and Care Research. (2024). New report on 10 promising AI interventions for healthcare. Available at: https://www.nihr.ac.uk/news/new-report-10-promising-ai-interventions-healthcare

[ref114] National Institute of Health. (2024a). Advancing Health Research through ethical, multimodal AI | data science at NIH. Available online at: https://datascience.nih.gov/artificial-intelligence/MultimodalAI (Accessed August 24, 2024).

[ref115] National Institute of Health. (2024b). Artificial intelligence at NIH | data science at NIH, Available online at: https://datascience.nih.gov/artificial-intelligence (Accessed May 31, 2024)

[ref116] National Institutes of Health. (2019). National Institutes of Health (NIH) — All of us. Available online at: https://allofus.nih.gov/ (Accessed October 07, 2023)

[ref117] National Institutes of Health, AIM-AHEAD. Data science at NIH. Available online at: https://datascience.nih.gov/artificial-intelligence/aim-ahead (Accessed May 31, 2024).

[ref118] National Science and Technology Council. (2022). National Strategy for Advanced Manufacturing a report by the Subcommittee on Advanced Manufacturing. Available online at: https://www.whitehouse.gov/wp-content/uploads/2022/10/National-Strategy-for-Advanced-Manufacturing-10072022.pdf (Accessed December 07, 2024)

[ref119] NCQA. (2018). Health plan accreditation (HPA) - NCQA. NCQA. Available online at: https://www.ncqa.org/programs/health-plans/health-plan-accreditation-hpa/ (Accessed December 16, 2024).

[ref120] NHS Beta. AI and digital regulations service for health and social care - AI regulation service - NHS. https://www.digitalregulations.innovation.nhs.uk/ (Accessed July 10, 2023).

[ref121] NHS England. (2017). Health survey for England - NHS digital. Available online at: https://digital.nhs.uk/data-and-information/publications/statistical/health-survey-for-england (Accessed August 29, 2024).

[ref122] NHS England. (2019). The NHS patient safety strategy safer culture, safer systems, safer patients NHS England and NHS improvement. Available online at: https://www.england.nhs.uk/wp-content/uploads/2020/08/190708_Patient_Safety_Strategy_for_website_v4.pdf (Accessed June 11, 2023)

[ref123] NHS England. (2019). Clinical audit. Available online at: https://www.england.nhs.uk/clinaudit/ (Accessed August 23, 2024).

[ref124] NHS England (2020). WE ARE THE NHS: People plan 2020/21 - action for us all. London, United Kingdom: NHS England.

[ref125] NHS England. (2021b). NHS England population health and the population health management Programme. Available online at: www.england.nhs.uk and https://www.england.nhs.uk/integratedcare/what-is-integrated-care/phm/ (Accessed December 12, 2022)

[ref126] NHS England. (2021a). NHS England integrated care systems: guidance. Available online at: www.england.nhs.uk and https://www.england.nhs.uk/publication/integrated-care-systems-guidance/ (Accessed September 05, 2024)

[ref127] NHS England. (2022). NHS England accelerating genomic medicine in the NHS Available online at: https://www.england.nhs.uk/publication/accelerating-genomic-medicine-in-the-nhs/ (Accessed August 10, 2023)

[ref128] NHS England. (2024). The generation study — knowledge hub. GeNotes. Available online at: https://www.genomicseducation.hee.nhs.uk/genotes/knowledge-hub/the-generation-study/ (Accessed April 24, 2024).

[ref129] NHS England. (n.d.a). Patient safety incident response framework. Available online at: https://www.england.nhs.uk/patient-safety/incident-response-framework/ (Accessed January 22, 2023)

[ref130] NHS England. (n.d.b). Learn from patient safety events (LFPSE) service. Available online at: https://www.england.nhs.uk/patient-safety/learn-from-patient-safety-events-service/ (Accessed January 22, 2023).

[ref131] NHSE. (2023). The human phenotype ontology — knowledge hub. GeNotes. Available online at: https://www.genomicseducation.hee.nhs.uk/genotes/knowledge-hub/the-human-phenotype-ontology/ (Accessed July 27, 2024)

[ref132] NHSE. (2024). NHS England» guidance on developing a 10-year infrastructure strategy. www.england.nhs.uk and https://www.england.nhs.uk/long-read/guidance-on-developing-a-10-year-infrastructure-strategy/ (Accessed August 01, 2024)

[ref133] NIH. (2024). Partnered research studies | all of us research program | NIH. All of us research program | NIH Available online at: https://allofus.nih.gov/funding-and-program-partners/partnered-research-studies (Accessed December 09, 2024).

[ref134] NIST. (2024). US AISI 1 and UK AISI 2 joint pre-deployment test. Available online at: https://www.nist.gov/system/files/documents/2024/11/19/Upgraded%20Sonnet-Publication-US.pdf (Accessed December 07, 2024)

[ref135] Obeidat O. Charles K. Akhter N. Tong A. T. (2023). Social risk factors that increase cardiovascular and breast Cancer risk. Curr. Cardiol. Rep. 25, 1269–1280. doi: 10.1007/s11886-023-01957-9, PMID: 37801282 PMC10651549

[ref136] OCR. (2008). Standards for privacy of individually identifiable health info. Available online at: https://www.hhs.gov/hipaa/for-professionals/privacy/guidance/standards-privacy-individually-identifiable-health-information/index.html (Accessed December 14, 2024).

[ref137] Office for National Statistics. (2023). Child and infant mortality in England and Wales - Office for National Statistics. Available online at: https://www.ons.gov.uk/peoplepopulationandcommunity/birthsdeathsandmarriages/deaths/bulletins/childhoodinfantandperinatalmortalityinenglandandwales/2021 (Accessed August 27, 2024)

[ref138] Office of Disease Prevention and Health Promotion. (2023). Social determinants of health - healthy people 2030. Available online at: https://health.gov/healthypeople/priority-areas/social-determinants-health#:~:text=Social%20determinants%20of%20health%20 (Accessed August 23, 2024)

[ref139] Office of Disease Prevention and Health Promotion. (2024). Healthy people 2030. Available online at: Health.gov and https://odphp.health.gov/healthypeople (Accessed December 09, 2024)

[ref141] ONS. (2024). National Life Tables – life expectancy in the UK - Office for National Statistics. Office for National Statistics, Available online at: https://www.ons.gov.uk/peoplepopulationandcommunity/birthsdeathsandmarriages/lifeexpectancies/bulletins/nationallifetablesunitedkingdom/2020to2022 (Accessed August 27, 2024).

[ref142] Our Future Health. (2021). Our future health. Available online at: https://ourfuturehealth.org.uk/

[ref143] Our Future Health and NHS. (2022). The UK’S largest ever health research programme to transform the prevention, detection and treatment of diseases – our future health. Available online at: https://ourfuturehealth.org.uk/the-uks-largest-ever-health-research-programme-to-transform-the-prevention-detection-and-treatment-of-diseases/ (Accessed June 05, 2022).

[ref144] Oxford Academic Press (2024). Opportunities and challenges for genomic data analyses in biobanks: a call for papers. Genetics 226. doi: 10.1093/genetics/iyae023, PMID: 40584554

[ref145] Park Y.-J. Pillai A. Deng J. Guo E. Gupta M. Paget M.. (2024). Assessing the research landscape and clinical utility of large language models: a scoping review. BMC Med. Inform. Decis. Mak. 24:72. doi: 10.1186/s12911-024-02459-6, PMID: 38475802 PMC10936025

[ref146] Patra B. G. Sharma M. M. Vekaria V. Adekkanattu P. Patterson O. V. Glicksberg B.. (2021). Extracting social determinants of health from electronic health records using natural language processing: a systematic review. J. Am. Med. Inform. Assoc. 28, 2716–2727. doi: 10.1093/jamia/ocab170, PMID: 34613399 PMC8633615

[ref147] Petrić Howe N. Bundell S. (2023). ‘Pangenome’ aims to capture the breadth of human diversity. Nature. doi: 10.1038/d41586-023-01579-9, PMID: 37165225

[ref148] Pimperl A. (2018). Re-orienting the model of care: towards accountable care organizations. Int. J. Integr. Care 18:15. doi: 10.5334/ijic.4162, PMID: 30127684 PMC6095061

[ref149] Population Health Analytics Laboratory. (2024). Artificial intelligence and population health. Population Health Analytics Laboratory. Toronto, ON

[ref150] Reddy S. (2024). Generative AI in healthcare: an implementation science informed translational path on application, integration and governance. Implement. Sci. 19:27. doi: 10.1186/s13012-024-01357-9, PMID: 38491544 PMC10941464

[ref151] Royal College of Pathologists. (2022). A new international standard: the introduction of ISO 15189:2022, www.rcpath.org, 2022. Available online at: https://www.rcpath.org/discover-pathology/news/a-new-international-standard-the-introduction-of-iso-15189-2022.html#:~:text=Jointly%20published%20with%20the%20Institute%20of%20Biomedical%20Science (Accessed March 11, 2023)

[ref152] Rumbold J. M. M. Pierscionek B. K. (2017). A critique of the regulation of data science in healthcare research in the European Union. BMC Med. Ethics 18. doi: 10.1186/s12910-017-0184-y, PMID: 28388916 PMC5385067

[ref153] Sabrin Afroz N. Habib I. M. A. Reza M. S. Alam M. A. (2024). Multi-omics data integration and drug screening of AML cancer using generative adversarial network. Methods 226, 138–150. doi: 10.1016/j.ymeth.2024.04.017, PMID: 38670415

[ref154] Sandhu S. Sharma A. Cholera R. Bettger J. P. (2021). Integrated health and social Care in the United States: a decade of policy Progress. Int. J. Integr. Care 21:9. doi: 10.5334/ijic.5687, PMID: 34785994 PMC8570194

[ref155] Sarvamangala D. R. Kulkarni R. V. (2021). Convolutional neural networks in medical image understanding: a survey. Evol. Intel. 15, 1–22. doi: 10.1007/s12065-020-00540-3, PMID: 33425040 PMC7778711

[ref156] Sarwar E. (2023). Clinical significance of precision medicine – genomics and pharmacogenomics (PGx). Advancing Global Bioethics 19, 33–54. doi: 10.1007/978-3-031-28593-6_3

[ref157] Schumacher G. J. Sawaya S. Nelson D. Hansen A. J. (2020). Genetic information insecurity as state of the art. Front. Bioeng. Biotechnol. 8:591980. doi: 10.3389/fbioe.2020.591980, PMID: 33381496 PMC7768984

[ref158] Scott R. H. Fowler T. A. Caulfield M. (2019). Genomic medicine: time for health-care transformation. Lancet 394, 454–456. doi: 10.1016/s0140-6736(19)31796-9, PMID: 31395438

[ref159] Shokrollahi Y. Yarmohammadtoosky S. Nikahd M. M. Dong P. Li X. Gu L. (2023). Comprehensive review of generative AI in healthcare. arXiv (Cornell University). doi: 10.48550/arxiv.2310.00795

[ref160] Siegel B. Erickson J. Milstein B. Pritchard K. E. (2018). Multisector partnerships need further development to fulfill aspirations for transforming regional health and well-being. Health Aff. 37, 30–37. doi: 10.1377/hlthaff.2017.1118, PMID: 29309220

[ref161] Singhal K. Azizi S. Tu T. Mahdavi S. S. Wei J. Chung H. W.. (2023). Large language models encode clinical knowledge. Nature 620, 172–180. doi: 10.1038/s41586-023-06291-2, PMID: 37438534 PMC10396962

[ref162] Song C. Ma Y. Xu Y. Chen H. (2024). Multi-population evolutionary neural architecture search with stacked generalization. Neurocomputing 587:127664. doi: 10.1016/j.neucom.2024.127664

[ref163] Spanos S. (2024). Healthcare leaders navigating complexity: a scoping review of key trends in future roles and competencies. BMC Med. Educ. 24:720. doi: 10.1186/s12909-024-05689-4, PMID: 38961343 PMC11223336

[ref164] Stackzone. (2024). A Deep Dive into Horizontal vs Vertical Scalability. Available online at: https://stackzone.com/post/horizontal-vs-vertical-scalability/ (Accessed November 24, 2024).

[ref165] Sun Y. Zhu C. Zheng S. Zhang K. Sun L. Shui Z.. (2024). PathAsst: a generative foundation AI assistant towards artificial general intelligence of pathology. Proceed AAAI Conf. Artif. Intelligence 38, 5034–5042. doi: 10.1609/aaai.v38i5.28308

[ref166] Tang Z. Chen G. Yang H. Zhong W. Chen C. Y.-C. (2024). DSIL-DDI: a domain-invariant substructure interaction learning for generalizable drug–drug interaction prediction. IEEE Transact. Neural Networks Learn Syst 35, 10552–10560. doi: 10.1109/tnnls.2023.3242656, PMID: 37022856

[ref167] Team DataMotion. (2019). HIPAA compliance in the age of population health management | data motion. DataMotion Available online at: https://datamotion.com/hipaa-compliance-in-the-age-of-population-health-management/ (Accessed December 14, 2024).

[ref168] The AI and digital regulations service, The AI and digital regulations service. NHS Transformation Directorate. Available online at: https://transform.england.nhs.uk/ai-lab/ai-lab-programmes/regulating-the-ai-ecosystem/the-ai-and-digital-regulations-service/ (Accessed July 28, 2024)

[ref169] The Joint Commission. (2024). The joint commission. Available online https://www.jointcommission.org/ (Accessed December 16, 2024).

[ref170] The Royal College of Pathologists. (2020). The haematology laboratory workforce: Challenges and solutions a meeting pathology demand briefing. Available online at: https://www.rcpath.org/static/4632d8bc-0451-4983-9639768129f758ec/haematology-laboratory-workforce-challenges-solutions.pdf (Accessed July 05, 2024)

[ref171] The White House. (2023). Executive order on the safe, secure, and trustworthy development and use of artificial intelligence. The White House, Available online at: https://www.whitehouse.gov/briefing-room/presidential-actions/2023/10/30/executive-order-on-the-safe-secure-and-trustworthy-development-and-use-of-artificial-intelligence/ (Accessed December 09, 2024).

[ref172] Tommel J. Kenis D. Lambrechts N. Brohet R. M. Swysen J. Mollen L.. (2023). Personal genomes in practice: exploring citizen and healthcare professionals’ perspectives on personalized genomic medicine and personal health data spaces using a mixed-methods design. Genes 14:786. doi: 10.3390/genes14040786, PMID: 37107544 PMC10137790

[ref173] Toufiq M. Rinchai D. Bettacchioli E. Kabeer B. S. A. Khan T. Subba B.. (2023). Harnessing large language models (LLMs) for candidate gene prioritization and selection. J. Transl. Med. 21. doi: 10.1186/s12967-023-04576-8, PMID: 37845713 PMC10580627

[ref174] Transformation Directorate, NHS AI Lab roadmap. NHS Transformation Directorate. Available online at: https://transform.england.nhs.uk/ai-lab/nhs-ai-lab-roadmap/ (Accessed May 31, 2024).

[ref175] U. Environment. (2021). GOAL 8: Decent work and economic growth. UNEP - UN Environment Programme. Available online at: https://www.unep.org/topics/sustainable-development-goals/why-do-sustainable-development-goals-matter/goal-8-decent-1 (Accessed November 21, 2024)

[ref176] U.S. Department of Health and Human Services. (2022). Summary of the HIPAA privacy rule. Available online at: https://www.hhs.gov/hipaa/for-professionals/privacy/laws-regulations/index.html (Accessed December 14, 2024).

[ref177] UK Government. (2021). National AI strategy. Available online at: https://www.gov.uk/government/publications/national-ai-strategy (Accessed December 09, 2024).

[ref178] UK Health Data Research. (2024). UKHDRA. Available online at: https://ukhealthdata.org/ (Accessed August 29, 2024).

[ref179] UK Health Data Research Alliance. (2024). UKHDRA. Available online at: ukhealthdata.org and https://ukhealthdata.org/ (Accessed July. 04, 2024)

[ref180] UK Parliament. (2024). Data (use and access) bill [HL] - parliamentary bills-UK parliament. Available online at; https://bills.parliament.uk/bills/3825 (Accessed December 07, 2024)

[ref181] UK.GOV. (2024). Collaboration on the safety of AI: UK-US memorandum of understanding. Available online at: https://www.gov.uk/government/publications/collaboration-on-the-safety-of-ai-uk-us-memorandum-of-understanding/collaboration-on-the-safety-of-ai-uk-us-memorandum-of-understanding?ref=ai-ethics.kr (Accessed May 30, 2024)

[ref182] United Nations. (2023). Goal 3: ensure healthy lives and promote well-being for all at all ages. United Nations. Available online at: https://sdgs.un.org/goals/goal3 (Accessed November 21, 2024)

[ref183] United Nations, ITU-WHO focus group on artificial intelligence for health (FG-AI4H) | Department of Economic and Social Affairs. Available online at: https://sdgs.un.org/partnerships/itu-who-focus-group-artificial-intelligence-health-fg-ai4h (Accessed May 28, 2024).

[ref184] US Department of Commerce. (2024). U.S. and UK announce partnership on science of AI safety. U.S. Department of Commerce, https://www.commerce.gov/news/press-releases/2024/04/us-and-uk-announce-partnership-science-ai-safety?form=MG0AV3 (Accessed Dec. 08, 2024).

[ref185] Vaught J. (2021). Biobanking best practices and publication standards. Singapore: Springer, 93–105.

[ref186] Wachter R. M. Brynjolfsson E. (2023). Will generative artificial intelligence deliver on its promise in health care? JAMA 331, 65–69. doi: 10.1001/jama.2023.25054, PMID: 38032660

[ref187] Wallace E. Lowry J. Smith S. M. Fahey T. (2013). The epidemiology of malpractice claims in primary care: a systematic review. BMJ Open 3:e002929. doi: 10.1136/bmjopen-2013-002929, PMID: 23869100 PMC3693415

[ref188] Wang S.. (2024). sdw95927/pathology-images-analysis-using-CNN. Available online at: https://github.com/sdw95927/pathology-images-analysis-using-CNN (Accessed May 15, 2024)

[ref189] Wang Y. Liu J. (2024). A comprehensive review of quantum machine learning: from NISQ to fault tolerance. Rep. Prog. Phys. 87, –116402. doi: 10.1088/1361-6633/ad7f69, PMID: 39321817

[ref190] Wang P. Vienneau M. Vogeli C. Schiavoni K. Jubelt L. Mendu M. L. (2023). Reframing value-based care management. JAMA Health Forum 4:e231502. doi: 10.1001/jamahealthforum.2023.1502, PMID: 37327007

[ref191] Wise J. (2024). Clinical trials: new UK framework aims to streamline process and improve transparency. BMJ 387:q2812. doi: 10.1136/bmj.q2812, PMID: 39672559

[ref192] Wood R. M. Budiman T. A. Hassey N. Onen Dumlu Z. Vasilakis C. Budd F. J.. (2023). Development and practical use of a risk-sensitive population segmentation model for healthcare service planning: Application in England. Int. J. Healthc. Manag. 1–10. doi: 10.1080/20479700.2023.2232980

[ref193] Woodward A. Urbanowicz R. J. Naj A. C. Moore J. H. (2022). Genetic heterogeneity: challenges, impacts, and methods through an associative lens. Genet. Epidemiol. 46, 555–571. doi: 10.1002/gepi.22497, PMID: 35924480 PMC9669229

[ref194] World Health Organization. (2022). International classification of diseases (ICD). Available online at: https://www.who.int/standards/classifications/classification-of-diseases (Accessed December 18, 2024).

[ref195] World Health Organization. (2023). Tracking universal health coverage: 2023 global monitoring report. Available online at: www.who.int https://www.who.int/publications/i/item/9789240080379 (Accessed December 9, 2024).

[ref196] Yang R. Tan T. R. Lu W. Thirunavukarasu A. J. Shu D. Liu N. (2023). Large language models in health care: development, applications, and challenges. Health Care Sci. 2, 255–263. doi: 10.1002/hcs2.61, PMID: 38939520 PMC11080827

[ref197] Yurkovich J. T. Evans S. J. Rappaport N. Boore J. L. Lovejoy J. C. Price N. D.. (2023). The transition from genomics to phenomics in personalized population health. Nat. Rev. Genet. 25, 286–302. doi: 10.1038/s41576-023-00674-x, PMID: 38093095

[ref198] Zhang S. Tong H. Xu J. Maciejewski R. (2019). Graph convolutional networks: a comprehensive review. Comput. Soc. Netw. 6:11. doi: 10.1186/s40649-019-0069-y, PMID: 37915858 PMC10615927

[ref199] Ziems C. Held W. A. Omar Ahmed Shaikh J. Chen Z. Z. Yang D. (2023). Can large language models transform computational social science? arXiv (Cornell University). doi: 10.48550/arxiv.2305.03514

[ref200] Zoph B. Le Q. V. (2017). “Neural architecture search with reinforcement learning,” arXiv preprint. arXiv:1611.01578. doi: 10.48550/arXiv.1611.01578

